# The assessment of epigenetic diversity, differentiation, and structure in the ‘Fuji’ mutation line implicates roles of epigenetic modification in the occurrence of different mutant groups as well as spontaneous mutants

**DOI:** 10.1371/journal.pone.0235073

**Published:** 2020-06-25

**Authors:** Xiaoyun Du, Yanbo Wang, Minxiao Liu, Xueqing Liu, Zhongwu Jiang, Lingling Zhao, Yan Tang, Yanxia Sun, Xueyong Zhang, Daliang Liu, Laiqing Song

**Affiliations:** 1 Yantai Academy of Agricultural Sciences, Yantai, Shandong, China; 2 Yantai University, Yantai, Shandong, China; Institute of Mediterranean Forest Ecosystems of Athens, GREECE

## Abstract

The ‘Fuji’ line includes many varieties with a similar genetic background and consistent inducement factors with epigenetic occurrence, thus it may be considered an ideal candidate for epigenetic research. In this study, 91 bud mutations of ‘Fuji’ apple were used as the test materials. Using the genetic variation within ‘Fuji’ as the control, the characteristics of epigenetic variation at different levels in both varieties and mutant groups were examined. The results showed that: (1) the global genomic DNA methylation level of the 91 bud mutants of ‘Fuji’ ranged from 29.120%-45.084%, with an average of 35.910%. Internal cytosine methylation was the main DNA methylation pattern. Regarding the variation of methylation patterns of ‘Fuji’ mutants, the vast majority of loci maintained the original methylation pattern existed in ‘Fuji’. CHG methylation variation was the main type of variation; (2) the variation in methylation patterns between the mutant groups was greater than that of methylation levels. Among these patterns, the variation in CHG methylation patterns (including CHG hypermethylation and CHG demethylation) was expected to be dominant. The observed variation in methylation levels was more important in the Color mutant group; however, the variation in methylation patterns was more obvious in both the early maturation and Spur mutant groups. Moreover, the range of variation in the Early-maturation group was much wider than that in the Spur mutant group; (3) epigenetic diversity and genetic diversity were both low between the mutant groups. In the ‘Fuji’ mutant groups, there was few correlation between genetic and epigenetic variation, and epigenetic differentiation resulted in more loci with moderate or greater differentiation; (4) the purifying selection seemed to play a major role in the differentiation of different groups of ‘Fuji’ mutants (65.618%), but epigenetic diversity selection still occurred at nearly 35% of loci. Sixteen epigenetic outlier loci were detected.

## Introduction

*Malus domestica* Borkh. cv. ‘Fuji’ is one of the most economically important apple varieties in China and the world. ‘Fuji’ apple is prone to mutation, through which abundant bud mutants have been derived, the majority of which have been adapted as superior varieties in production [1]. Studies have shown that epigenetics is an important cause of bud mutations in fruit crops, but it has not yet to be systematically studied in a large sample size. The study of the characteristics of epigenetic effects in bud mutants is the premise for further studying the epigenetic mechanism of mutations and carrying out epigenetic breeding.

Epigenetics studies the heritable changes in gene function that eventually lead to phenotypic variation with no changes in the underlying DNA sequence [2]. Epigenetics is involved in the regulation of gene expression, and changes are dynamic with respect to the endogenous and/or external environmental stimuli, thus affecting the phenotypic plasticity and environmental adaptability of organisms [3]. Epigenetic variation enhances biodiversity and complexity, especially in asexual organisms without gene recombination, which is helpful to the promotion of functional phenotypic diversity and has a greater impact on variation and evolution and more survival significance [4].

DNA methylation, representing the most important form of epigenetic modification, is ubiquitous in higher plants [5]. Different level of DNA methylation exists in normal organisms (5%-40%, [6]), while changes in DNA methylation can be caused by external environmental factors or various biotic and abiotic stresses [7]. Changes in DNA methylation level and methylation pattern may cause significant phenotypic variation in plant genome [8]. It can be expressed by the dynamics of hypermethylation and demethylation [9]. DNA methylation occurs on cytosines in different sequence contexts, and CG and CHG are the two main types of genome methylation. CG and CHG methylation are regulated by distinct enzymes and pathways [10]. Both CG and CHG methylation levels are different in a plant species, and CG methylation was reported predominately in many plant species [11].

The current research on DNA methylation is mainly focused on local and global aspects. The former mainly targets specific traits, starting with the changes directly related gene methylation in promoter or gene core sequence, and has more advantages in revealing the mechanism of occurrence of specific phenotypic variation; The latter studies the changes in the overall methylation of the genome, which is more conducive to the comprehensive analysis of the role of epigenetics in the variation and diversity. Many techniques have been developed to analyze global DNA methylation and its alterations [12]. The method of methylation-sensitive amplified polymorphism (MSAP) is one of the most efficient, economical, and widely one used for the detection of DNA methylation events [13–14]. This approach is a modified version of the amplified fragment length polymorphism (AFLP) method based on the differential sensitivity of isoschizomeric restriction endonucleases to site-specific cytosine methylation [15]. MSAP analysis employs two types of cleaved enzymes *Eco*RI (rare cutter) and *Hpa*II/*Msp*I (frequent cutters) which recognize similar tetranucleotide 5’-CCGC representing a different sensitivity to methylation at the inner or outer cytosine. If one or both cytosines are methylated at both DNA strands, *Hpa*II is inactive. If one or both cytosines are methylated in only one strand they are cleaved by *Hpa*II. In contrast, *Msp*I reacts when only the internal cytosine is hemi- or fully- (double strand) methylated [16]. So, on the basis of variant band patterns resulting from differential digestion of the genome by *Hpa*II/*Msp*I isozymes, variation in the DNA methylation level and pattern in the whole genome is detected [15, 17]. MSAP has been successfully applied for the analysis of the variation in methylation levels and patterns in a variety of plant species [13].

In general, bud mutations are an important source of new varieties of fruit crops. Characteristics such as a perennial nature, long juvenile phase, heterozygosity, and sexual incompatibilities in fruit crops hamper their improvement through conventional breeding [18]. Compared with conventional hybridization, the selection of bud mutants gives the advantages of shortening the breeding cycle and reducing the workload and costs. Such an approach can be used to obtain excellent varieties by modifying individual traits without changing the desirable qualities of the parent plant [19]. A variety of perennial fruit trees of economic importance originated from bud changes [20–21]. However, Spontaneous bud mutation occurs at a very low frequency, moreover, many of these mutations may be deleterious, making the organism less adapted to its environment, and in some cases may even be lethal [18]. Therefore, mutants that survive in adverse environments and even present excellent phenotypes are considered to have good characteristics for adaption. Epigenetic regulation mediated by DNA methylation is considered as one of the important mechanisms in plant adaptive procedure [14, 22]. Studying the molecular mechanism of bud mutation at the DNA level is thus of great significance.

‘Fuji’ is the most representative apple cultivar in which plenty of new bud mutations arise [23]. Multiple bud mutants generated from the standard cultivar ‘Fuji’. Those represent highly similar genetic backgrounds and have abundant types of variation. Additionally, ‘Fuji’ bud mutants were frequently reported to be available in orchards at unusual locations, such as under dense high-voltage lines, at high altitudes, subjected to an abnormal climate, severe drought, waterlogging, frost, or sudden diseases or insect pest infestations [24]. This means that the conditions for induction of bud mutation are consistent with the factors inducing epigenetic effects. Therefore, in a summary of the above mentioned, ‘Fuji’ mutation line could be regarded as ideal sets for research on epigenetic regulation. However, to our knowledge, global DNA methylation in this line has not been reported yet.

A general understanding of the mechanisms of genome-wide DNA methylation in ‘Fuji’ mutants is a prerequisite for their utilization in epigenetic mechanism studies or breeding. Therefore, in the present study, nearly one hundred ‘Fuji’ bud mutants were used as study materials for the first time. Then, through genetic variation as control, we focused on the analysis of variations in DNA methylation levels and patterns within groups and between groups as well as epigenetic diversity.

We were interested in the following questions: (1) is DNA methylation involved in the occurrence of bud mutations in the ‘Fuji’ line? (2) what are the characteristics of the changes in both DNA methylation levels and patterns? (3) what are the effects of DNA methylation on not only the clustering status of the ‘Fuji’ line at the variety level but also genetic structure and differentiation at the group level? (4) how is natural selection affecting the occurrence of different mutant groups in the Fuji line?

## Materials and methods

### Plant material and MSAP, AFLP analyses

This study was conducted on ‘Fuji’ and it’s 91 elite varieties arising from bud mutations. Three major types: Color mutant group, Early-maturation mutant group, and Spur mutant group could be classified (Table 1). We sampled plant materials with the same age on the same dates (12 June 2016) and at their identical phenological stage (bearing fully expanded leaves), trying to avoid that possible developmental variation in DNA methylation would confound variety or group differences in methylation patterns. Fully expanded fresh leaves from pooled individuals (ca. 5 plants being clonally propagated from a single mother variety) of each variety were collected. Young leaves immediately frozen in liquid nitrogen and then stored at -80°C prior to DNA isolation.

**Table 1:** Plant materials and summary of MSAP amplification in this study.

Variety Code	Name	Mutation type	Total loci	Non-methylated CCGG loci (%)	Methylated CCGG loci (%)		
					CG-methylated	CHG- methylated	Total
M1	Fuji	Origin	1841	1212(65.834)	396(21.510)	233(12.656)	629(34.166)
M2	Shanfu No.6	Color	2112	1227(58.097)	402(19.034)	483(22.869)	885(41.903)
M3	Iwate line I	Color	1880	1134(60.319)	475(25.266)	271(14.415)	746(39.681)
M4	Iwafu No.10	Color	1904	1163(61.082)	478(25.105)	263(13.813)	741(38.918)
M5	Line I Fuji	Color	1996	1136(56.914)	552(27.655)	308(15.431)	860(43.086)
M6	Gunfu No.1	Color	1875	1268(67.627)	363(19.360)	244(13.013)	607(32.373)
M7	Aki Fuji	Color	1893	1231(65.029)	453(23.930)	209(11.041)	662(34.971)
M8	Lele Fuji	Color	1962	1222(62.283)	497(25.331)	243(12.385)	740(37.717)
M9	Aomori-fu No.13	Color	2025	1263(62.370)	363(17.926)	399(19.704)	762(37.630)
M10	2001	Color	1874	1231(65.688)	396(21.131)	247(13.180)	643(34.312)
M11	Aki-fu No.1	Color	1872	1238(66.132)	438(23.397)	196(10.470)	634(33.868)
M12	Aki-fu No.5	Color	1910	1270(66.492)	433(22.670)	207(10.838)	640(33.508)
M13	Morioka-fu No.1	Color	2018	1294(64.123)	363(17.988)	361(17.889)	724(35.877)
M14	Morioka-fu No.2	Color	1950	1289(66.103)	373(19.128)	288(14.769)	661(33.897)
M15	Tensei	Color	1989	1260(63.348)	471(23.680)	258(12.971)	729(36.652)
M16	Qingnonghe No.2	Color	1916	1288(67.223)	387(20.198)	241(12.578)	628(32.777)
M17	Qianxuan No.3	Color	1908	1263(66.195)	380(19.916)	265(13.889)	645(33.805)
M18	Fubrax	Color	1871	1231(65.794)	411(21.967)	229(12.239)	640(34.206)
M19	Nagafu No.12	Color	1939	1287(66.374)	353(18.205)	299(15.420)	652(33.626)
M20	Nagafu No.1	Color	1923	1266(65.835)	341(17.733)	316(16.433)	657(34.165)
M21	Nagafu No.2	Color	1998	1281(64.114)	406(20.320)	311(15.566)	717(35.886)
M22	Nagafu No.4	Color	1928	1272(65.975)	399(20.695)	257(13.330)	656(34.025)
M23	Nagafu No.6	Color	2026	1297(64.018)	372(18.361)	357(17.621)	729(35.982)
M24	Nagafu No.7	Color	1933	1322(68.391)	377(19.503)	234(12.106)	611(31.609)
M25	Nagafu No.36	Color	1904	1201(63.078)	464(24.370)	239(12.553)	703(36.922)
M26	Wengao No.1	Color	1871	1254(67.023)	379(20.257)	238(12.720)	617(32.977)
M27	Wengao-M No.1	Color	1968	1302(66.159)	450(22.866)	216(10.976)	666(33.841)
M28	Wengao No.2	Color	1884	1317(69.904)	332(17.622)	235(12.473)	567(30.096)
M29	Wengao-M No.2	Color	1917	1267(66.093)	358(18.675)	292(15.232)	650(33.907)
M30	Wengao-M No.3	Color	2066	1270(61.471)	438(21.200)	358(17.328)	796(38.529)
M31	Yanfu No.1	Color	1976	1233(62.399)	440(22.267)	303(15.334)	743(37.601)
M32	Yanfu No.2	Color	1940	1249(64.381)	457(23.557)	234(12.062)	691(35.619)
M33	Yanfu No.3	Color	1952	1278(65.471)	449(23.002)	225(11.527)	674(34.529)
M34	Yanfu No.4	Color	1962	1243(63.354)	429(21.865)	290(14.781)	719(36.646)
M35	Yanfu No.5	Color	2014	1300(64.548)	435(21.599)	279(13.853)	714(35.452)
M36	Yanfu No.8	Color	1915	1233(64.386)	445(23.238)	237(12.376)	682(35.614)
M37	Yanfu No.10	Color	1921	1267(65.955)	433(22.540)	221(11.504)	654(34.045)
M38	92–58	Color	2103	1383(65.763)	440(20.922)	280(13.314)	720(34.237)
M39	Meili	Color	1933	1298(67.150)	404(20.900)	231(11.950)	635(32.850)
M40	Meile	Color	2013	1272(63.189)	503(24.988)	238(11.823)	741(36.811)
M41	Tianfu No.1	Color	2004	1310(65.369)	385(19.212)	309(15.419)	694(34.631)
M42	Tianfu No.2	Color	1980	1270(64.141)	462(23.333)	248(12.525)	710(35.859)
M43	Yanchanghong	Color	1947	1259(64.664)	446(22.907)	242(12.429)	688(35.336)
M44	Zhaoyuanstripe red Fuji	Color	2021	1304(64.523)	458(22.662)	259(12.815)	717(35.477)
M45	Zhaoyuanflush red Fuji	Color	2136	1247(58.380)	610(28.558)	279(13.062)	889(41.620)
M46	Shoufu No.1	Color	2031	1281(63.072)	517(25.455)	233(11.472)	750(36.928)
M47	Shoufu No.2	Color	2039	1309(64.198)	518(25.405)	212(10.397)	730(35.802)
M48	Yulindian Fuji	Color	1978	1242(62.791)	493(24.924)	243(12.285)	736(37.209)
M49	Changfujia	Color	2035	1282(62.998)	426(20.934)	327(16.069)	753(37.002)
M50	Zhaofuwang	Color	1885	1290(68.435)	339(17.984)	256(13.581)	595(31.565)
M51	Yishuihong	Color	1893	1160(61.278)	439(23.191)	294(15.531)	733(38.722)
M52	Jihong	Color	1904	1259(66.124)	450(23.634)	195(10.242)	645(33.876)
M53	Yannongzaofu	Early-maturation	1793	1141(63.636)	423(23.592)	229(12.772)	652(36.364)
M54	Zaoshu Fuji	Early-maturation	1884	1116(59.236)	490(26.008)	278(14.756)	768(40.764)
M55	Early Fuji A	Early-maturation	1965	1203(61.221)	471(23.969)	291(14.809)	762(38.779)
M56	Hongjiangjun	Early-maturation	1862	1202(64.554)	411(22.073)	249(13.373)	660(35.446)
M57	Hongwangjiang	Early-maturation	1804	1222(67.738)	301(16.685)	281(15.576)	582(32.262)
M58	Jinfu No.1	Early-maturation	1927	1201(62.325)	491(25.480)	235(12.195)	726(37.675)
M59	Gai Fuji	Early-maturation	1851	1169(63.155)	405(21.880)	277(14.965)	682(36.845)
M60	Yuhuazaofu	Early-maturation	1816	1149(63.271)	391(21.531)	276(15.198)	667(36.729)
M61	Qianxuan No.1	Early-maturation	1847	1220(66.053)	408(22.090)	219(11.857)	627(33.947)
M62	Qianxuan No.2	Early-maturation	1889	1226(64.902)	450(23.822)	213(11.276)	663(35.098)
M63	Changhong	Early-maturation	1923	1231(64.015)	393(20.437)	299(15.549)	692(35.985)
M64	New ryoka	Early-maturation	1884	1219(64.703)	411(21.815)	254(13.482)	665(35.297)
M65	Ryoka	Early-maturation	1862	1165(62.567)	459(24.651)	238(12.782)	697(37.433)
M66	Jinfu No.2	Early-maturation	1855	1213(65.391)	405(21.833)	237(12.776)	642(34.609)
M67	Jinfu No.3	Early-maturation	1892	1217(64.323)	381(20.137)	294(15.539)	675(35.677)
M68	Yishuizhongqiu	Early-maturation	1892	1200(63.425)	448(23.679)	244(12.896)	692(36.575)
M69	Fengfuji No.1	Early-maturation	1888	1160(61.441)	452(23.941)	276(14.619)	728(38.559)
M70	Shoufu No.3	Early-maturation	1928	1232(63.900)	472(24.481)	224(11.618)	696(36.100)
M71	Changyanghong	Early-maturation	2057	1458(70.880)	340(16.529)	259(12.591)	599(29.120)
M72	Yiyuan Nagafu No.2	Early-maturation	1883	1161(61.657)	452(24.004)	270(14.339)	722(38.343)
M73	Hirosaki fuji	Early-maturation	1975	1181(59.797)	565(28.608)	229(11.595)	794(40.203)
M74	Karakida Fuji	Early-maturation	2120	1265(59.670)	497(23.443)	358(16.887)	855(40.330)
M75	Sufuji	Early-maturation	1867	1201(64.328)	381(20.407)	285(15.265)	666(35.672)
M76	Qiufuhong	Spur	2085	1145(54.916)	614(29.448)	326(15.635)	940(45.084)
M77	Yanfu No.6	Spur	1818	1235(67.932)	367(20.187)	216(11.881)	583(32.068)
M78	Yanfu No.7	Spur	1820	1194(65.604)	418(22.967)	208(11.429)	626(34.396)
M79	Huimin spur	Spur	1882	1219(64.772)	440(23.379)	223(11.849)	663(35.228)
M80	Duanzhi Fuji	Spur	1852	1222(65.983)	390(21.058)	240(12.959)	630(34.017)
M81	Miyazaki	Spur	1853	1200(64.760)	399(21.533)	254(13.708)	653(35.240)
M82	Tiaowen Gongqi	Spur	2000	1150(57.500)	583(29.150)	267(13.350)	850(42.500)
M83	Aki-fu No.39	Spur	1934	1182(61.117)	452(23.371)	300(15.512)	752(38.883)
M84	Longfu	Spur	1853	1198(64.652)	399(21.533)	256(13.815)	655(35.348)
M85	Fukushima spur	Spur	1883	1216(64.578)	408(21.668)	259(13.755)	667(35.422)
M86	Aomori spur	Spur	1884	1217(64.597)	461(24.469)	206(10.934)	667(35.403)
M87	Qinfu No.1	Spur	1941	1241(63.936)	445(22.926)	255(13.138)	700(36.064)
M88	Liquan spur	Spur	1840	1177(63.967)	411(22.337)	252(13.696)	663(36.033)
M89	Lingbaoduanfu	Spur	1842	1182(64.169)	440(23.887)	220(11.944)	660(35.831)
M90	Chengji No.1	Spur	1857	1189(64.028)	434(23.371)	234(12.601)	668(35.972)
M91	Yiyuanhong	Spur	1942	1252(64.470)	376(19.361)	314(16.169)	690(35.530)
M92	Shifu spur	Spur	1814	1187(65.436)	413(22.767)	214(11.797)	627(34.564)
**Total**	**Min**		1793	1116(54.916)	301(16.528)	195(10.242)	567(29.120)
	**Max**		2136	1458(70.880)	614(29.448)	483(22.870)	940(45.084)
	**Mean**		1930	1236(64.090)	431(22.311)	263(13.599)	694(35.910)

Total genomic DNA from leaf tissue was extracted by using DNeasy Plant Mini Kit (Qiagen). MSAP molecular marker was used for the analysis of epigenetic variation in plants examined. For comparison, genetic variation was also conducted by AFLP molecular marker. Both methods performed on the same set of plants, and *Eco*RI primers were labeled with or green (JOE), blue (FAM), or yellow (NED) fluorescent dyes.

MSAP and AFLP were performed as described by Xiong et al. [17] and Vos et al. [25], respectively. MSAP was essentially the same as the AFLP protocol. The difference between MSAP and AFLP procedure was replacing the *Mse*I enzyme with the enzyme either *Hpa*II or *Msp*I in MSAP. Thus, differences in the PCR products detected with *Eco*RI/*Hpa*II and *Eco*RI/*Msp*I would reflect different methylation states. The primer sequences of MSAP and AFLP were listed in the S1 Table. Based on previous pilot tests, we selected 23 optimal primer combinations for MSAP analysis (S2 Table) and 19 optimal primer combinations for the AFLP analysis (S3 Table). HM-, E-, and M- corresponded to the sequence of H/M00, E00, and M00, respectively (S1 Table). The repeatability of banding patterns assessed by conducting two sets of independent MSAP and AFLP analyses and only the consistent bands were included.

Using an automated sequencer (ABI PRISM^®^3730 Genetic Analyzer, Applied Biosystems) to separated and detected MSAP and AFLP PCR products. GeneScan Rox-500 labeled with a red (ROX) dye was an internal size marker. A peak size between 80 and 500 bp was selected to study the polymorphic DNA fragments. MSAP products were scored as present ‘1’ or absent ‘0’ on the chromatogram to create a binary matrix.

### Statistical analysis

The resulting data of MSAP and AFLP were processed using Excel 2016. According to the scoring approach [26], MSAP raw data were transformed into a binary data matrix before running statistical analyses and computation. Because the enzymes used for MSAP analysis recognize the same restriction site (5’-CCGG) but have different sensitivities to methylation modifications, the final products of MSAP present four types of DNA methylation at the 5’-CCGG sites, namely, no methylation (H1M1, condition I), CHG methylation (H1M0, condition II), CG methylation (H0M1, condition III), and CG/CHG methylation (H0M0, condition IV). DNA methylation level% = (condition II+condition III)/(condition I+condition II+condition III+condition IV).

Methylation-Sensitive Polymorphism (MSP) matrix data, which were converted using R program *msap* [27], were used for all analyses of DNA methylation. The change of patterns of cytosine methylation at CCGG sites in the ‘Fuji’ mutation line was listed in Table 2. Accordingly, contrasting to the standard cultivar ‘Fuji’, the changes of DNA methylation pattern in its series mutations could be summarized into four categories: (a) CG hyper: H1M1 to H0M1, H1M0 to H0M0, H1M0 to H0M1, H1M1 to H0M0; (b) CHG hyper: H1M1 to H1M0, H0M1 to H0M0, H1M1 to H0M0, H0M1 to H1M0; (c) CG hypo: H0M1 to H1M1, H0M0 to H1M0, H0M1 to H1M0; and (d) CHG hypo: H1M0 to H1M1, H0M0 to H0M1, H1M0 to H0M1, H0M0 to H1M1.

**Table 2:** Changes in the patterns of cytosine methylation at CCGG sites in the ‘Fuji’ mutation line.

Pattern		Band type digested by E+H /E+M		Pattern		Band type digested by E+H /E+M	
Type	Sub-type	Fuji	mutation	Type	Sub-type	Fuji	Mutation
A	A1	+/+	+/-	C	C1	-/+	+/-
	A2	+/+	+/+		C2	-/+	+/+
	A3	+/+	-/+		C3	-/+	-/+
	A4	+/+	-/-		C4	-/+	-/-
B	B1	+/-	+/-	D	D1	-/-	+/-
	B2	+/-	+/+		D2	-/-	+/+
	B3	+/-	-/+		D3	-/-	-/+
	B4	+/-	-/-				

(1) E: *Eco*R I enzyme; H: *Hpa*II enzyme; M: *Msp*I enzyme; (2) +: band present; -: band absent; +/+: band present in both E+H and E+M; +/-: band present in E+H but absent in E+M; -/+: band absent in E+H but present in E+M; -/-: band absent in both E+H and E+M.

### DNA methylation analysis and variation coefficient calculation of different mutant groups in ‘Fuji’

The epigenetic relationship of different mutant groups in ‘Fuji’ was analyzed with 12 indexes related to DNA methylation levels and patterns. Indicators include CHG hyper-methylation frequency (V5), CG hyper-methylation frequency (V6), CG hypo-methylation frequency (V7), CHG hypo-methylation frequency (V8), the total hyper-methylation frequency (V9), the total hypo-methylation frequency (V10), total genome methylation frequency (V11), the frequency of condition I (Non-methylation frequency, V12), the frequency of condition II methylation (CHG methylation frequency, V13), the frequency of condition III methylation (CG methylation ratio, V14), the frequency of condition IV methylation (V15), and the total amplified loci of varieties (V16).

The Coefficient of Variation (CV) of each of the above indexes was calculated in the three mutation groups in order to investigate whether varieties in different mutant groups behave in different ways. CV = (standard deviation SD/Mean)×100%. Correlation analysis of 12 major epigenetic parameters (V5-V15) was performed by software IBM SPSS Statistics 22 [28].

### Epigenetic and genetic similarity, clustering and principal component analysis

The similarity coefficient between varieties was calculated using the SM coefficient through the SimQual procedure of NTSYSpc2.11 software package [29] and unweighted pair group method average (UPGMA) method was used for cluster analysis. Circle diagrams were drawn using TBtools [30]. MSAP-PCA and AFLP-PCA analysis were carried out by R program package *msap* 27 and Adegenet 2.1.1 [31], respectively.

### Epigenetic and genetic diversity and molecular variation analysis

Various diversity measurement parameters including polymorphism site proportion (PPL), effective allele variance (Ne), Shannon diversity index (I), expected heterozygosity (He), and hierarchical analysis of molecular variance (AMOVA) analysis between and within mutant groups were estimated in GenAlex 6.51 [32], using 999 random permutations.

### Epigenetic and genetic Structural analysis

STRUCTURE 2.3.4 [33] was utilized to analyze genetic and epigenetic structure. Admixture and correlated allele frequencies model were chosen. Ten independent runs were made with values of K set from 2 to 4, with three iterations for each value of K. The length of the burn-in period was set at 10,000, and the number of Markov chain Monte Carlo (MCMC) repeats after burn-in was set at 100,000. The result from STRUCTURE output file was performed online by STRUCTRE Harvester 0.6.8 [34]. The results were averaged for a particular K using CLUMPP 1.1.2 [35] and visualized by DISTRUCT [36].

### Potential outlier detection

BAYESCAN v2.1 [37] was used to test Fst outliers in global and pairwise comparisons. A reversible-jump Markov chain Monte Carlo algorithm based on a Bayesian likelihood approach is used in BAYESCAN to estimate the ratio of posterior probabilities of selection over neutrality [the posterior odds (PO)]. Based on Jeffreys’ [38] scale of evidence, a log_10_PO>2.0 is interpreted as ‘strong evidence’ of selection. For our analysis, the estimation of model parameters was set as 10 pilot runs of 5,000 interaction each, followed by 100,000 interactions [37]. Outliers were calculated using a burn-in of 50,000 interactions, a thinning interval of 20, and a sample size of 5000. FDR = 0.05 was used.

### Correlation analysis between epigenetic diversity and genetic diversity

Using NTSYS2.0 software, the correlations between epigenetic distance and genetic distance of three ‘Fuji’ mutant groups as well as varieties calculated by MSAP and AFLP were evaluated using the Mantel test implemented through NTSYSpc 2.11 software package [28].

All of the statistical significance in this study was determined by IBM SPSS Statistics 22 [32]. A value of P < 0.05 was considered significant.

## Results

### MSAP and AFLP amplification

A total of 2954 CCGG loci were detected via the genome-wide methylation analysis of 92 ‘Fuji’ varieties with 23 pairs of MSAP primer combinations, and 129 CCGG loci were amplified on average with each pair of primers. Among these loci, 2752 CCGG loci showed polymorphism, for a polymorphic ratio of 93.162%. Different MSAP amplification patterns were obtained from different varieties (Table 1). The number of amplified methylated loci ranged from 1793 to 2136, and that of polymorphic loci ranged from 1613 to 1956, with an average polymorphic ratio of 63.566% (58.612% to 71.076%). Among the 2954 CCGG loci, 1748 were methylation-sensitive loci (MSL), accounting for 59.174% of the total amplified loci. A total of 1627 polymorphic MSL loci were obtained, accounting for 93.078% of the total amplified loci (S2 Table).

Nineteen pairs of AFLP primer combinations amplified 1745 total loci in the same test varieties used for MSAP, among which 1620 were polymorphic loci, accounting for 92.837% of the total amplified loci. Different primer combinations produced different amplification results. The total number of amplified loci obtained with a single pair of primers ranged from 36 to 166, the number of polymorphic loci ranged from 28 to 150, and the polymorphic ratio ranged from 57.143% to 100%. On average, the total numbers of loci and polymorphic loci generated by amplification with each pair of primers were 92 and 86, respectively. The four primer combinations (E-AGG+M-CAG, E-AGG+M-CTA, E-ACG+M-CAC, E-ACG+M-CTG) produced 100% polymorphic loci. Primer amplification details are shown in S3 Table.

### Analysis of DNA methylation levels and variation patterns at the variety level

As shown in Table 1, the global genomic DNA methylation level of the 91 varieties was 29.120%-45.084%, with an average of 35.929%. Among these modifications, the internal cytosine methylation level was 22.311% (16.528%-29.448%), and the external cytosine methylation level was 13.599% (10.242%-22.870%). The former was significantly higher than the latter (*P*<0.01), implying that internal cytosine methylation was the main DMA methylation way in the ‘Fuji’ line.

According to the cutting profiles of the *Hpa*II and *Msp*I methylation-sensitive endonucleases in the original ‘Fuji’ variety, the banding patterns could be divided into four types: A, B, C, and D (Table 2). In comparison with the original ‘Fuji’, there were many types of possible locus variation in the mutant varieties, so the variation pattern of methylation loci could be subdivided into several subcategories: A1, A2, A3, A4, B1, B2, B3, B4, C1, C2, C3, C4, D1, D2, and D3. As shown in S1 Fig., in DNA methylation variation patterns A and C, loci identical to the original methylation pattern presented the highest proportion, indicating that during the occurrence of bud mutations in ‘Fuji’ line, the majority of loci maintained the original methylation pattern, while only a few loci exhibited methylation variation (S5 Table). All types of methylation variation patterns (15 subclasses) were detected in the test varieties.

The above 15 subclasses of methylation variation patterns were further categorized into four types: CG hypermethylation (CG-hyper), CHG hypermethylation (CHG-hyper), CG demethylation (CG-hypo) and CHG demethylation (CHG-hypo). As shown in S4 Table, CHG-hypo, CHG-hyper, CG-hyper, and CG-hypo displayed differences among different tested varieties, but the general trend basically showed the following correlations: CHG-hypo>CHG-hyper>CG-hyper>CG-hypo (S2 Fig). (CHG-hypo+CHG-hyper) was significantly higher than (CG-hypo+CG-hyper) (*P*<0.01). The relative trend between the total demethylation frequency and the hypermethylation frequency in different varieties also exhibited diversity, including the following findings: (1) the demethylation frequency was higher than the frequency of methylation, (2) the hypermethylation frequency was higher than that of demethylation, and (3) hypermethylation and demethylation frequencies were approximately the same. These results indicated that methylation pattern variations were not fixed during the occurrence of the ‘Fuji’ mutation.

### Analysis of the DNA methylation level and variation pattern at the mutant group level

The epigenetic relationships of the genomes of different mutant groups in the ‘Fuji’ line were analyzed using 12 parameters related to DNA methylation levels and patterns (V5-V16). Table 3 showed that the variation coefficients (CVs) of V5, V6, V7, V8, V9, and V10 were generally greater than those of V11, V12, V13, V14, V15, and V16. Among these 12 parameters, V5-V10 and V11-V16 reflected variations in the DNA methylation pattern and DNA methylation level, respectively. Therefore, it could be deduced that the variation of methylation patterns among varieties was greater than that of methylation levels. V11-V16, V11, V12, V15, and V16 exhibited the same degree of variation among groups, while V13 and V14 showed relatively higher CV than these groups, indicating that variation in CHG and CG methylation levels is abundant among varieties of different mutant groups in the ‘Fuji’ line.

**Table 3:** Variations in the levels of 12 DNA methylation parameters and pattern-related parameters in three ‘Fuji’ mutant groups.

Parameter	Std.Deviation			Mean			CV (%)		
	Spur	Early-maturation	Color	Spur	Early-maturation	Color	Spur	Early-maturation	Color
**V5**	2.033	2.776	1.802	12.722	13.571	14.608	15.977	20.454	12.338
**V6**	1.412	2.436	1.896	9.833	10.796	13.463	14.363	22.563	14.081
**V7**	1.965	2.558	2.732	6.682	7.346	10.223	29.401	34.817	26.726
**V8**	3.239	2.466	2.846	14.265	14.977	18.743	22.703	16.464	15.182
**V9**	2.848	4.946	3.342	22.555	24.367	28.072	12.626	20.299	11.907
**V10**	3.691	4.267	3.462	20.947	22.324	28.966	17.622	19.115	11.953
**V11**	3.141	2.616	2.605	36.328	36.427	35.572	8.645	7.181	7.324
**V12**	3.141	2.616	2.605	63.672	63.573	64.428	4.932	4.115	4.044
**V13**	1.523	1.583	2.464	13.187	13.77	13.679	11.548	11.497	18.016
**V14**	2.668	2.734	2.675	23.142	22.656	21.894	11.531	12.068	12.218
**V15**	2.444	2.552	2.260	35.334	34.989	32.948	6.917	7.293	6.854
**V16**	72.196	75.503	66.710	1889	1898	1959	3.823	3.978	3.406

V5, CHG hyper-methylation frequency; V6, CG hyper-methylation frequency; V7, CG hypo-methylation frequency; V8, CHG hyper-methylation frequency; V9, the total hyper-methylation frequency; V10, the total hypo-methylation frequency; V11, total genome methylation frequency; V12, the frequency of condition I (Non-methylation frequency); V13, the frequency of condition II methylation (CHG methylation frequency); V14, the frequency of condition III methylation (CG methylation ratio); V15, the frequency of condition IV methylation; V16, the total amplified loci of varieties.

The CVs of each parameter were compared between the three mutant groups and analyzed (Table 3). It was shown that the emphases of epigenetic variation were different in different groups; for example, in the Early-maturation group, several parameters, including V5, V6, V7, V9, and V10, presented higher values than in the other groups; in the Spur group, V8 was much more prominent; in the Color group, V13 showed a remarkably high value. Based on the above results, it seemed that the variation in the methylation level was more important in the Color group. The variation in the methylation pattern was obvious in the Early-maturation and Spur groups; moreover, the extent of the variation in the Early-maturation group was much wider.

**Fig 1:**
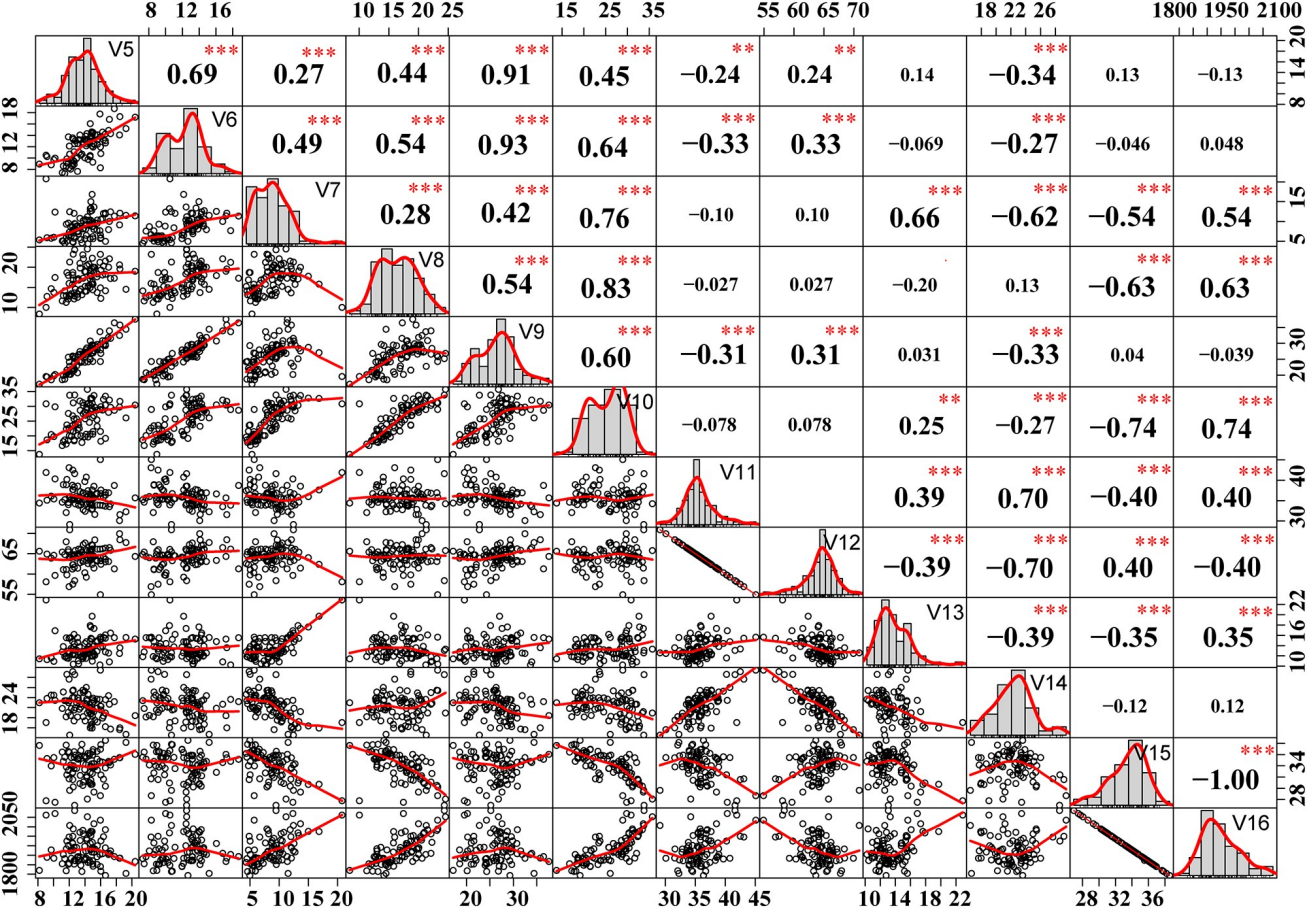
Correlation analysis of 12 epigenetic parameters. Three asterisks represent a very significant correlation (P < 0. 01). The data distribution is displayed on the diagonal of the matrix; the bivariate scatter with the fitting line is displayed in the lower left; the correlation coefficient and the significance level are displayed in the upper right.

The correlations of 12 DNA methylation parameters were calculated and plotted using the R packages *psych* and *corrplot*. As presented in Fig 1, a total of 43 pairs (*P*<0.01) were significantly correlated with each other, among which V6 and V9 exhibited the highest significant positive correlation (0.93), followed by V5 and V9 (0.91). The lowest correlation was found for V10 and V13 (0.25), followed by V9 and V12 (0.31). The significant negative correlation was highest between V5 and V11 (-0.24), followed by V10 and V14 (-0.27). The lowest negative correlation was found for V15 and V16 (-1.00), followed by V10 and V15 (-0.74). The results showed that there was a direct positive correlation between the total hypermethylation frequency and the frequency of CG hypermethylation (that is, the higher the frequency of CG hypermethylation, the higher the frequency of total hypermethylation). The total demethylation frequency presented a small correlation with the proportion of CHG methylation but a significant negative correlation with the proportion of CG methylation (that is, the higher the proportion of CG methylation, the lower the total demethylation frequency).

**Fig 2:**
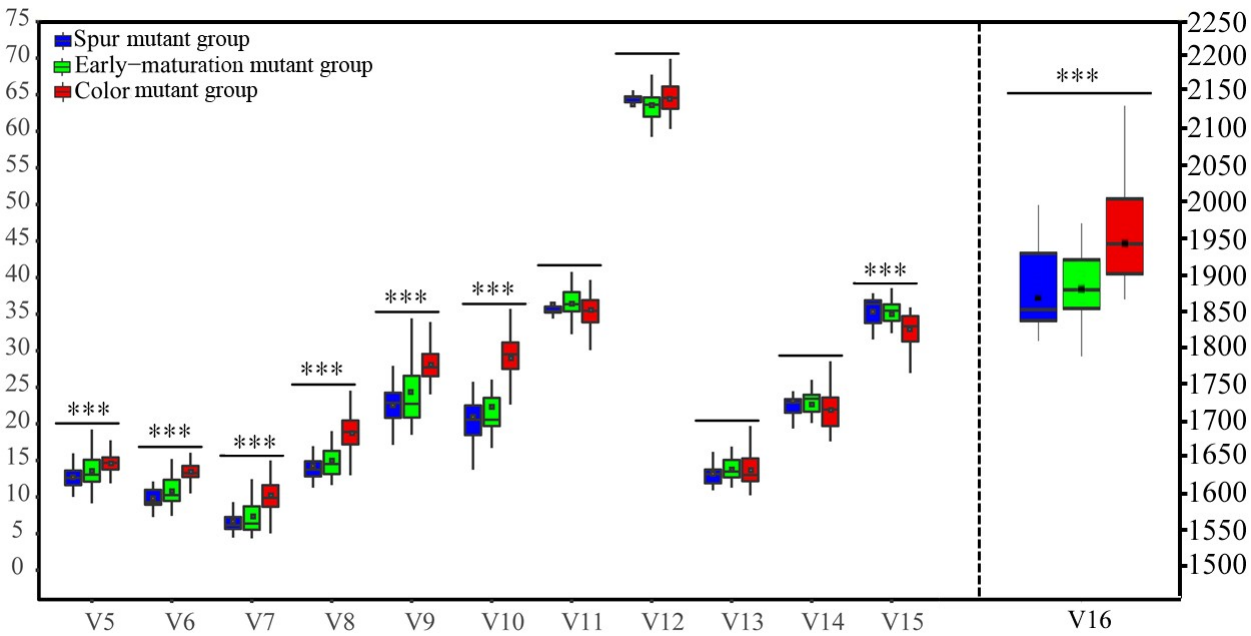
AMOVA of 12 epigenetic parameters between different mutant groups in the ‘Fuji’ mutation line. Blue, green, and red indicate the materials from the Spur group, the Early-maturation group, and the Color mutant group, respectively.

Furthermore, the above 12 DNA methylation parameters of the three mutant groups were compared and analyzed. The data distribution of all 12 variables conformed to a normal distribution and the assumptions of the statistical analysis. The variance homogeneity test showed that, with the exception of V9 (the total hypermethylation frequency), the other 11 parameters all met the requirements of homogeneity of variance. Therefore, the Tamhane's T2 and Games-Howell tests were chosen for V9, whereas the Duncan and LSD tests were chosen for the other parameters for multiple comparison analysis with SPSS software. Multiple comparative analysis among groups showed that among the three mutant groups, V5, V6, V7, V8, V9, V10, V15, and V16 presented significant differences (*P*<0.01), but no significant difference was found between V11, V12, V13, and V14. The average multiple comparison results revealed that the differences between the Spur and Early-maturation groups were not significant, showing similar epigenetic characteristics, but different degrees of significant differences existed between the Spur and Color mutant groups as well as between the Early-maturation and Color groups. For example, V5, V6, V7, V8, V9, V10, V15, and V16 were significantly different between the Spur and Color mutant groups; the difference in V6, V7, V8, V9, V10, V15, and V16 between the Early-maturation and Color mutant groups was very significant (Fig 2). Taken together, the above results showed that among the three ‘Fuji’ mutant groups, the Spur and Early-maturation groups showed similar epigenetic patterns; however, between the Color groups and either the Spur group or the Early-maturation group, epigenetic differences were apparent.

### Epigenetic similarity calculation, clustering and principal component analysis

The results of the genetic similarity analysis based on the MSAP-MSL data sets (S6 Table) showed that the epigenetic similarity coefficient between varieties ranged from 0.515 to 0.807; the lowest similarity was found between ‘Yishuizhongqiu’ and ‘Wengao No.3’, and the highest similarity existed between ‘Longfu’ and ‘Liquan spur’. The average epigenetic similarity between varieties in the ‘Fuji’ mutation line was 0.639, ranging from 0.577–0.678. The mean similarity coefficient between the standard ‘Fuji’ variety and its descendant varieties was 0.648, ranging from 0.561 (between ‘Fuji’ and ‘Zhaoyuanflush red Fuji’) to 0.758 (between ‘Fuji’ and ‘Yanfu No.7’). The results showed that there was apparently epigenetic variation between mutant varieties and their ‘Fuji’ mother, and different degrees of epigenetic differentiation also occurred between different mutant varieties.

**Fig 3:**
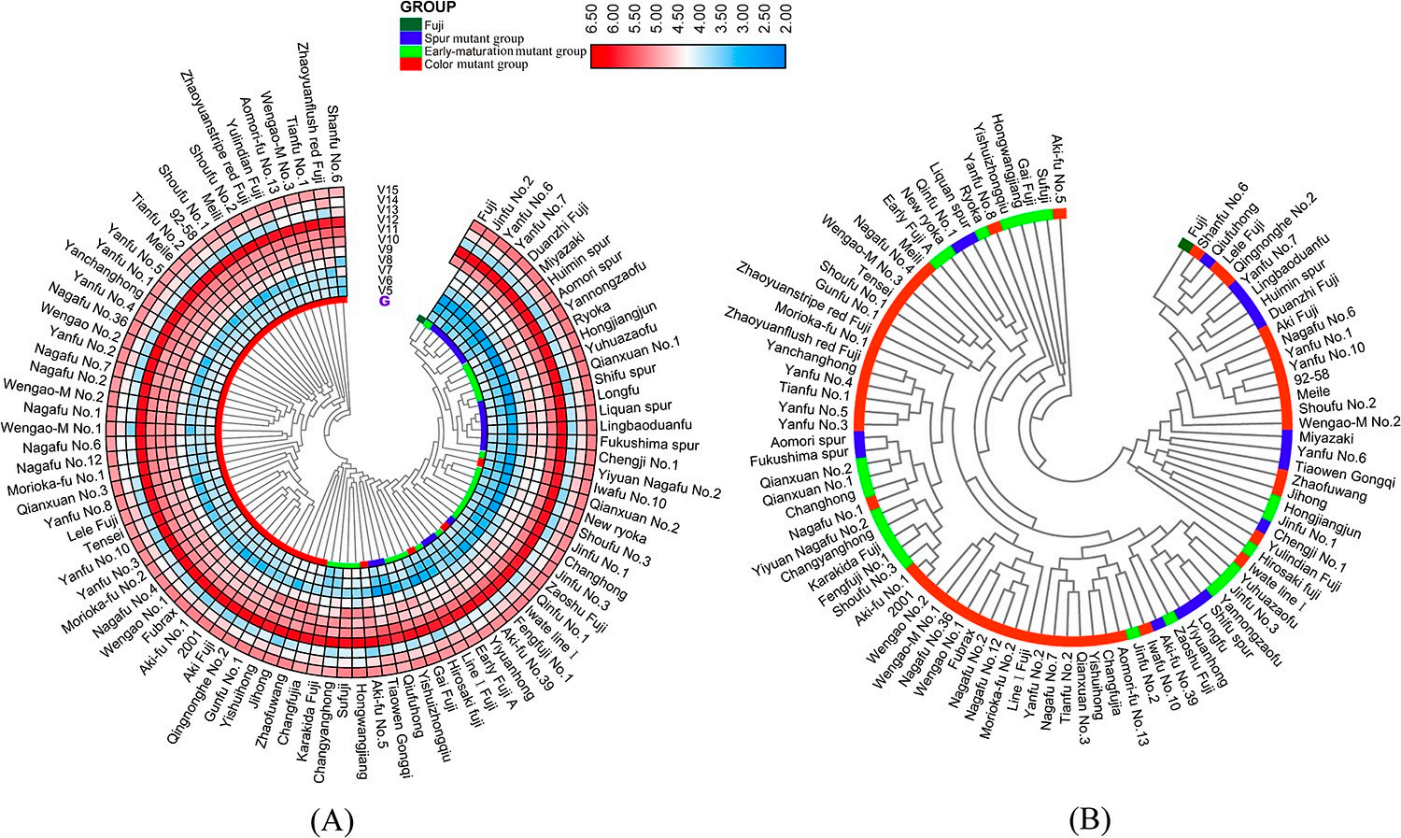
Dendrogram of 92 varieties obtained from UPGMA cluster analysis based on MSAP-MSL (A) and AFLP (B) data and the distribution of 16 methylation parameters of V5-V15 in the heat map between samples. Blue, green, and red in the circle of interest indicate the materials from the Spur group, the Early-maturation group, and the Color group, respectively.

The UPGMA results showed (Fig 3(A)) that nearly 84% (43/51) of the varieties in the Color mutant group clustered closely together to form an independent cluster (Cluster 1). The Early-maturation mutant group, the Spur-maturation mutant group, ‘Fuji’, and the remaining eight varieties of the Color mutant group formed another group, designated Cluster 2. In comparison with the position of ‘Fuji’, other varieties showed obvious epigenetic variation, and the epigenetic variation of the Color mutant group was most prominent. We also found that within Cluster 2, the Early-maturation mutant group and Spur mutant group were mixed and distributed without distinct grouping, reflecting the close epigenetic relationship between them. In addition, although ‘Chang Fujia’, ‘Zhaofuwang’, ‘Yishuihong’, and ‘Jihong’ in the Color mutant group were concentrated in Cluster 2, they were relatively closer to Cluster 1, but ‘Aki-fu No.5’, ‘Line I Fuji’, ‘Iwate line I’, and ‘Iwafu No.10’ were located far from Cluster 1. These four varieties were all selected in Japan and belonged to the Color mutant group. Moreover, they were the earliest mutant cultivars obtained from ‘Fuji’, and few clonal descendants are selected from these cultivars at present. Therefore, their clustering results might be related to their original region of selection or breeding generation. In conclusion, the UPGMA results implied that the greatest epigenetic variation existed in the Color mutant group, which might present unique epigenetic and evolutionary mechanisms compared with the other two variant groups.

**Fig 4:**
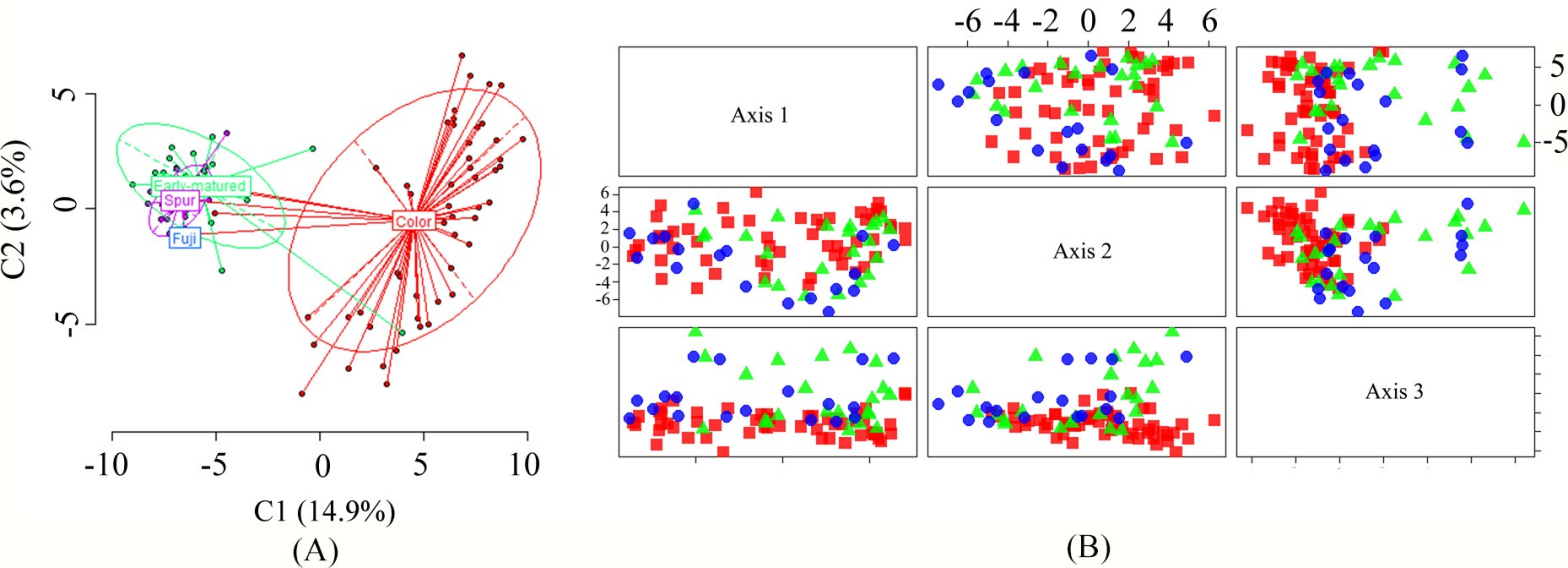
Principal coordinate analysis (PCA) of 92 varieties based on MSAP-MSL (A) and AFLP (B). Blue, green, and red indicate the materials from the Spur group, the Early-maturation group, and the Color group, respectively.

The results of principal component analysis further supported the UPGMA clustering results. Principal components PC1 and PC2 accounted for 13.6% and 4.5% of the total variation, respectively. As shown in Fig 4(A), the Spur mutant and Early-maturation groups were mixed, showing similar epigenetic consistency. ‘Fuji’ was included in this group, displaying a close epigenetic relationship with its members. The Color mutant group was basically independent and was located far from ‘Fuji’ and the other two mutant groups, indicating its unique epigenetic variation.

### Genetic similarity calculation, clustering and principal component analysis

Genetic similarities of 0.653 (between ‘Miyazaki’ and ‘Su Fuji’) to 0.899 (between ‘Nagafu No.12’ and ‘Nagafu No.2’) were detected by NTSYS in the ‘Fuji’ mutation line, with an average similarity of 0.805 (S7 Table). The mean similarity coefficient between the standard ‘Fuji’ variety and its mutants was 0.80, ranging from 0.684 (between ‘Fuji’ and ‘Su Fuji’) to 0.877 (between ‘Fuji’ and ‘Yanfu No.7’), indicating that the genetic variation of the mutants differed from that of ‘Fuji”.

The UPGMA results showed (Fig 3(B)) that 92 samples could be clustered into four groups (Clusters 1–4), and ‘Aki-fu No.5’ was clustered in the outermost area and separated into different groups, indicating distant genetic similarity. In addition, ‘Hongwangjiang’, ‘Gai Fuji’, and ‘Su Fuji’ were grouped together to form Cluster 3. Cluster 1 and Cluster 2 were closely related to each other. ‘Fuji’ was clustered in Cluster 1, which was closely related to ‘Shanfu No.6’, ‘Qiufuhong’, ‘Lele Fuji’, and ‘Qingnonghe No.1’. In Cluster 1 and Cluster 2, local clustering and accumulation phenomena of the same ‘Fuji’ mutation type were found, as observed in the regions between ‘Yanfu No.7’ and ‘Duanzhi Fuji’ (Spur mutant group), ‘Aki Fuji’ and ‘Nagafu No.36’ (Color mutant group), and ‘Miyazaki’ and ‘Chengji No.1’ (Spur mutant group) in the former group and the region between ‘Qianxuan No.1’ and ‘Shoufu No.3’ and that between ‘Yanfu No.3’ and ‘Nagafu No.4’ in the latter group. However, from the overall perspective of the cluster diagram, the three major ‘Fuji’ mutant groups were mixed and arranged, and there was no obvious boundary between different groups. Fig 4(B) showed the PCA results of the 92 test varieties. The three dimensions of PC1 and PC2, PC1 and PC3, and PC2 and PC3 all showed results that were consistent with UPGMA clustering; that is, the genetic similarity among the three major mutation types of ‘Fuji’ was very high, without obvious boundaries.

### Epigenetic and genetic diversity and molecular variation analysis

As shown in Table 4, the mean Shannon index, the number of effective alleles, and the mean expected heterozygosity based on MSL data among the three mutant groups were 0.415, 1.424, and 0.266, respectively. In general, the three genetic diversity indexes showed a consistent variation trend. For mutants with a high Shannon index (I), the effective allelic variance (Ne) and mean expected heterozygosity (He) were also higher. The I and He indices presented significant differences among the three mutant groups (*P*<0.01). The analysis of molecular variance showed that most of the variation occurred within the mutant groups (85%), and only a very small proportion (15%) occurred among the mutant groups (*P<* 0.01).

**Table 4:** Epigenetic and genetic similarity and diversity in three different mutant groups of the ‘Fuji’ line.

Molecular marker type	Group	Percentage of Polymorphic Loci (%)	Epigenetic/Genetic diversity parameter		
			I	Ne	He
**MSAP**	Spur	82.895	0.388A	1.404A	0.250A
	Early-maturation	92.677	0.413B	1.428AB	0.266B
	Color	99.542	0.443C	1.440B	0.282C
	Mean	91.705	0.415	1.424	0.266
**AFLP**	Spur	60.516	0.284a	1.300a	0.183a
	Early-maturation	70.029	0.289a	1.307a	0.186a
	Color	80.573	0.296a	1.303a	0.188a
	Mean	70.372	0.290	1.303	0.186

Abbreviations: Ne, No. of effective alleles; I, Shannon index; He, Expected heterozygosity. Lower case letters indicate a significant difference at the 0.05 level. Lowercase letters indicate a significant difference at the 0.01 level.

The average Shannon index, number of effective alleles and average expected degree of heterozygosity in the three ‘Fuji’ mutant groups reflected by AFLP analysis were 0.290, 1.303 and 0.186, respectively. In general, there was no significant difference in genetic diversity indexes between the three groups basing on AFLP data. AMOVA showed that most of the genetic variation existed within the mutant groups (95%); a very small portion (5%) existed between the populations (*P*< 0.01).

### Epigenetic and genetic structure analysis

**Fig 5:**
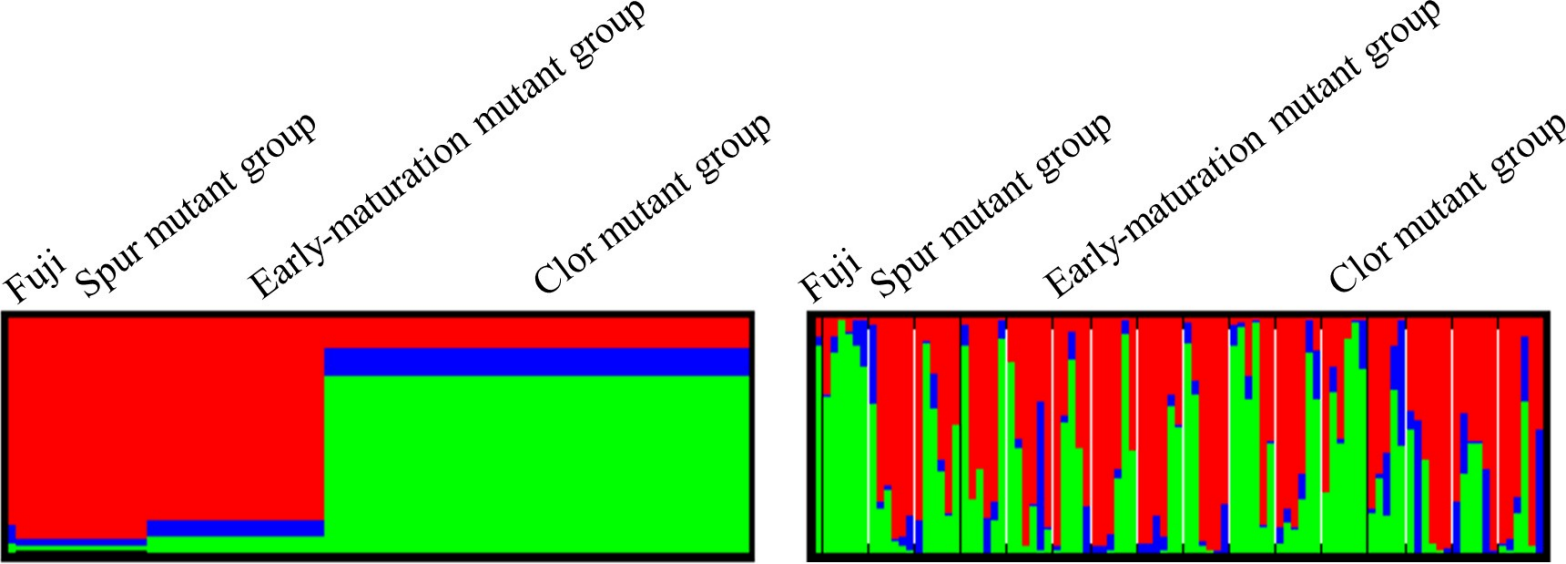
Population structure based on MSAP-MSL (A) and AFLP (B) analysis for ‘Fuji’ and its mutants.

Structure analysis was carried out based on MSL epigenetic data and AFLP-based genetic data, and 10,000 simulations were run with population numbers K = 2 to 4. The results showed that LnP (D) values were generated for K = 3 for both types of data, and no values were generated for K = 2 or K = 4. Therefore, K = 3 was selected as the optimal number of clusters. Fig 5(A) showed that in the ‘Fuji’ mutation line, the Spur mutant group and the Early-maturation mutant group were highly consistent, while the Color mutant group showed a large proportion of heteromorphic lineages, indicating the unique epigenetic structure of the Color mutant group. As shown in Fig 5(B), there was no clear division between any of the mutant groups, which reflected the similarity of their genetic compositions.

### Outlier analysis

**Fig 6:**
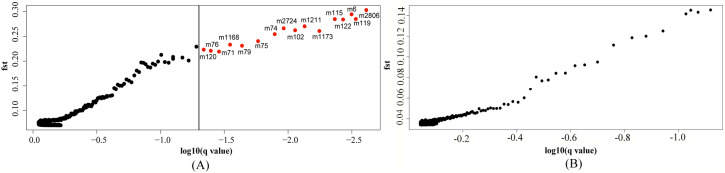
Bayesian genomic scans of the three mutant groups in the ‘Fuji’ line to identify outlier loci based on MSAP-MSL (A) and AFLP data (B).

**Fig 7:**
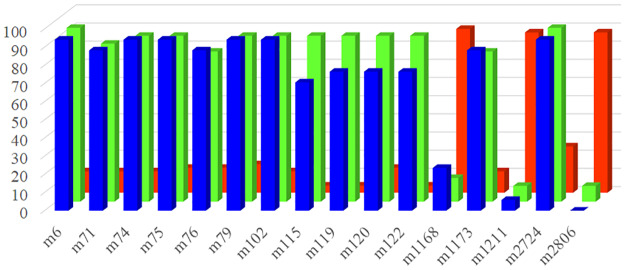
The frequency of 16 outlier loci in 3 different groups of the ‘Fuji’ mutation line based on the MSAP-MSL assay. Blue, green, and red indicate the materials from the Spur group, the Early-maturation group, and the Color group, respectively.

Bayesian population genomic analysis showed that out of 1748 methylation-sensitive MSAP loci, 601 exhibited positive alpha values, and 1147 exhibited negative alpha values. A positive alpha value indicates positive or diversified selection, while negative values indicate the negative or purifying selection, also known as neutral selection. Thus, purifying selection seemed to play a major role in the differentiation of the different groups of ‘Fuji’ mutants (65.618%). However, epigenetic diversity selection still occurred at nearly 35% of loci. Sixteen epigenetic outlier loci were detected, accounting for 0.915% of the total DNA methylation-sensitive loci (Fig 6(A)). The epigenetic genetic frequencies of each locus in each mutant group were calculated individually. If the frequency of a locus in a group was greater than 50%, it was set as a specific outlier locus of that population. Therefore, Fig 7 showed that there were 13, 13, and 3 methylated outlier loci in the Spur, Early-maturation, and Color mutant groups, respectively. According to the profile of the frequency of loci, the Spur and Early-maturation mutant groups presented the same pattern, which basically maintained the state of the existence of the majority of loci in ‘Fuji’. However, the pattern of the Color mutant group was completely different from that of the other two mutant groups as well as ‘Fuji’. In the Color mutant group, 13 locus deletions and 3 new locus mutations occurred, indicating different epigenetic patterns of the outlier loci among the different mutant groups and suggesting unique epigenetic characteristics of the Color mutant group.

According to AFLP analysis, among the 1745 amplified loci, Bayesian population genomic analysis generated 568 loci with positive alpha values and 1177 loci with negative values. Purifying selection played a major role (67.450%) in the differentiation of the different ‘Fuji’ mutant groups. Genetic diversity selection occurred at only a few loci (32.550%). At the level of FDR = 0.05, the number of outlier loci detected was 0, indicating that there was no significant difference in genetic differentiation between the three ‘Fuji’ mutant groups (Fig 6(B)).

### Mantel correlation test of epigenetic and genetic similarity between different mutant groups

The Mantel test was conducted for the genetic and epigenetic similarity coefficient matrix obtained based on MSAP and AFLP analysis. The results showed that the r values of the genetic and epigenetic correlation coefficients of the Spur mutant group, Early-maturation mutant group, and Color mutant groups were -0.044 (P = 0.368), 0.327 (P = 0.978), and 0.107 (P = 0.924), respectively. The overall correlation coefficient was 0.108 (P = 0.987). The above results showed that there was a slight correlation between genetic and epigenetic variation in the ‘Fuji’ mutation lines, suggesting that the epigenetic variation was independent of genetic variation.

### Comparison of epigenetic and genetic diversity between different mutant groups

The average polymorphic loci ratio, the polymorphic loci ratio, as well as epigenetic/genetic diversity parameters of each ‘Fuji’ mutant group based on MSAP were significantly higher than those obtained based on AFLP analysis (*P*<0.01), indicating that the epigenetic diversity of each mutant group in the ‘Fuji’ mutation line was significantly higher than the genetic diversity. For AMOVA between as well as within the mutant group, a certain degree of differentiation under both markers was revealed, and among groups, the epigenetic differentiation ratio (15%) was greater than the genetic differentiation ratio (5%).

### Comparison of epigenetic and genetic structure between different mutant groups

The results of the UPGMA clustering analysis, PCA, and structure analysis based on AFLP and MSAP data showed that the three ‘Fuji’ mutant groups were genetically similar and that, regardless of what algorithm the analysis was based on, they could not be clearly separated. In terms of epigenetics, there was a close relationship between the Spur and the Early-maturation mutant groups, which were always mixed, similar to their genetic relationship. However, for the varieties of the Color mutant group tended to cluster together, and this group was independent of the other two mutant groups, showing its unique epigenetic structure.

### Comparison of outlier loci between different mutant groups

The numbers of loci with positive and negative alpha values were approximately the same when generated on the basis of AFLP and MSAP. For each marker type, the proportion of loci with a negative alpha value was greater than that with a positive alpha value. Regarding outlier loci, MSAP analysis detected 16 loci, whereas AFLP analysis detected 0, indicating that epigenetics played a large role in the differentiation of the ‘Fuji’ mutant groups; and from the viewpoint of the frequency of outlier loci between different groups, epigenetics in the Color mutant group contributed greatly to the differentiation of the ‘Fuji’ mutant groups.

## Discussion

Extensive epigenetic research has been carried out in many plant species, whereas study on the fruit crops is still in progress. So far, the available reports mainly consist of several popular fruit crops such as apple [39–48], orange [49–50], banana [51], pear [52–53], strawberry [54], and grape [55–56]. Epigenetic studies in bud mutations are just in the initial stages since limited literature is available [40–41, 45–47, 52–53]. In ‘Fuji’ apple bud mutation occurrence, epigenetic mechanisms mediated by DNA methylation begun only last year [46–48], and all of them were done from the perspective of local DNA methylation. Few studies were performed on global DNA methylation in bud variation with a large sample size even on fruit crops. In the present study, we have incorporated a large-scale collection of ‘Fuji’ mutants from around the world as plant materials. Moreover, investigations of the genetic and epigenetic perspectives, at both the individual variety and mutant groups were conducted for the first time. Our studies revealed wide DNA methylation alterations between bud mutations in the ‘Fuji’ line. The results would be helpful for comprehensively understanding the epigenetic characteristics and differentiation of the ‘Fuji’ line. It also benefits for future further research on the epigenetic mechanism of bud mutations.

From an adaptive perspective, the modification of methylation status may allow trees to rapidly respond to abrupt changes in environmental conditions and contribute to their long-term responses to more general environmental scenarios [57]. Bud mutations often occur under stresses such as drought, pathogen infection, extreme weather and climate conditions, limited nutrient availability, human activities, and natural or artificial selection and are a result of adaptation to abnormal external environmental conditions [58, 59]. The contribution of epigenetic modification to the ability of plants to adapt to various stresses has been well demonstrated [26, 22]. Therefore, it can be theoretically deduced that a certain mutation is associated with epigenetic variation to some extent. This study detected nearly 32% epigenetic differentiation between the mutants and their original mother ‘Fuji’, showing many types of methylation pattern variants, and indicating that the variation in DNA methylation might be involved in the occurrence of ‘Fuji’ mutations. Our results explain the differentiation of epi-phenotypes, related to the similar genome structure, highlighted by Guarino et al. [60]. This may also verify the hypothesis of significant plant promotion to environmental adaptation, by phenotype diversification, increasing the probability of the plant to thrive when faced with a changing environment [3].

Different levels and degrees of methylation occur in higher plants to maintain normal plant development [59]. In general, the total genome DNA methylation level in different species detected by MSAP analysis is between 4.7% and 60.0% [61]. In this research, the DNA methylation level detected by MSAP was approximately 36%, similar to the findings of Li [62] in apple, in accordance with the relatively stable characteristics of DNA methylation levels in this species.

CG and CHG are two main forms of DNA methylation at CCGG sites. Their relative proportions in the organism vary among different species, and the majority of available reports present results show that the proportion of the former is greater than of the latter [11]. In this study, the levels of CG and CHG methylation were found to be 22.311% and 13.599%, respectively. The level of the former was significantly higher than that of the latter, indicating full methylation derived was the main form of DNA methylation in the ‘Fuji’ line, which conformed to the results in the majority of plant species.

Natural mutations in plants are usually accompanied by changes in the levels of genetic and epigenetic. AFLPs and MSAPs are the two marker techniques commonly used for the detection of plant genetic and epigenetic variation [13–14]. Expected heterozygosity (He), also known as gene diversity [63], is an important parameter for the measurement of the general genetic diversity among plant groups and shows wide applicability to any system of polyploidy, self-crossing or asexual reproduction in populations [64]. The lower the genetic diversity, the higher the degree of the genetic homogeneity in a population. It is generally known that a heterozygosity value higher than 0.5 indicates genetic diversity between population individuals [65]. In this study, the average genetic diversity values of the two types of molecular markers were 0.186 (AFLP) and 0.266 (MSAP), suggesting that the genetic and epigenetic diversity levels of the mutant groups in the ‘Fuji’ line were relatively low. Comparatively, in the three examined mutant groups, the epigenetic expected heterozygosity of the Color mutant group was higher than those of the other two groups (*P*<0.01), implying its relatively high epigenetic diversity. Additionally, in combination with the results from UPGMA cluster, STRUCTURE analysis, and outlier loci detection, the Color mutant group displayed unique epigenetic characteristics, which contributed largely to the epigenetic differentiation between the three mutant groups in the ‘Fuji’ line. This phenomenon might be related to the degree of difficulty in bud mutation of certain traits, the breeding and selection objectives in a given period, or the degree of artificial intervention.

Epigenetic variations may exhibit phenotypic differences in different environments or growth periods and even in different tissues and plant organs [61]. However, in some species such as walnut, the global genomic DNA methylation level in different tissues and organs does not differ significantly [65]. The leaf can provide information about epigenetic modification and adaptation in response to different environmental conditions [60]. In our study, the leaves were considered as the plant material, as such, the results and conclusions of the epigenetic investigation were limited to the analysis of DNA matrix purified from particular tissue collection. The differences between the Early-maturation mutant group and the Color mutant group were mainly reflected in the performance of fruits. Therefore, if fruit samples of these two mutant types were used as the examined material, the results would not be similar to those obtained for leaves and remain inconclusive since no similar research has previously been conducted in apple. The tissue specificity of epigenetic modifications is also unknown.

Additionally, we found that the mean hypermethylation and demethylation frequencies of CHG type were both significantly higher than those of CG type, indicating that variation in CHG methylation pattern played a key role in DNA methylation pattern variation in the ‘Fuji’ lines. In plants, cytosine methylation is a context-dependent process [9]. Our findings supported it. DNA methylation status reflects the outcome of the dynamic regulation of establishment, maintenance, and active-removal activities. CG and CHG methylation are catalyzed by various different enzymes and are under control by different pathways [9]. Hence, in view of our findings, the mechanism of epigenetic involvement in the occurrence of ‘Fuji’ bud mutation could be further explored, focusing on the act of enzymes and detailed pathways related to CHG methylation.

In conclusion, the present study uncovered abundant changes in methylation levels and patterns between not only bud mutants and their mother ‘Fuji’ but also bud mutants, indicating that it may be possible that epigenetics mediated by DNA methylation was involved in the occurrence of the ‘Fuji’ bud mutation line. The epigenetic mechanism of the Color bud mutant group was unique, which could be the focus of further research. The correlation between epigenetic variation and genetic variation was weak, showing their independence, which provided further support for using ‘Fuji’ line as ideal sets for epigenetic studies in the future.

## Supporting information

S1 Fig Variation patterns of 15 subclasses of DNA methylation patterns. (TIF)

S2 Fig Summary of the changes in the cytosine methylation pattern in the ‘Fuji’ mutation line compared with the standard ‘Fuji’ cultivar. (TIF)

S1 Table Adaptors and primer sequences used for preamplification in AFLP and MSAP analyses. (DOCX)

S2 Table Summary of MSAP amplification from 23 primer combinations. (DOCX)

S3 Table Summary of AFLP amplification in 92 materials. (DOCX)

S4 Table Variation of the four major types of DNA methylation patterns (CG hypermethylation, CHG hypermethylation, CG hypomethylation, and CHG hypomethylation) in the ‘Fuji’ mutants. (DOCX)

S5 Table Number and frequency of change in cytosine methylation pattern in the bud sports compared with the standard ‘Fuji’. (XLSX)

S6 Table Genetic similarity based on MSAP-MSL profiles. (XLSX)

S7 Table Genetic similarity based on AFLP data profiles. (XLSX)
