# Supplementary figures and images for: The assessment of epigenetic diversity, differentiation, and structure in the ‘Fuji’ mutation line implicates roles of epigenetic modification in the occurrence of different mutant groups as well as spontaneous mutants
Source: PLoS One. 2020 Jun 25;15(6):e0235073. doi: 10.1371/journal.pone.0235073 (PMC7316255; doi:10.1371/journal.pone.0235073)

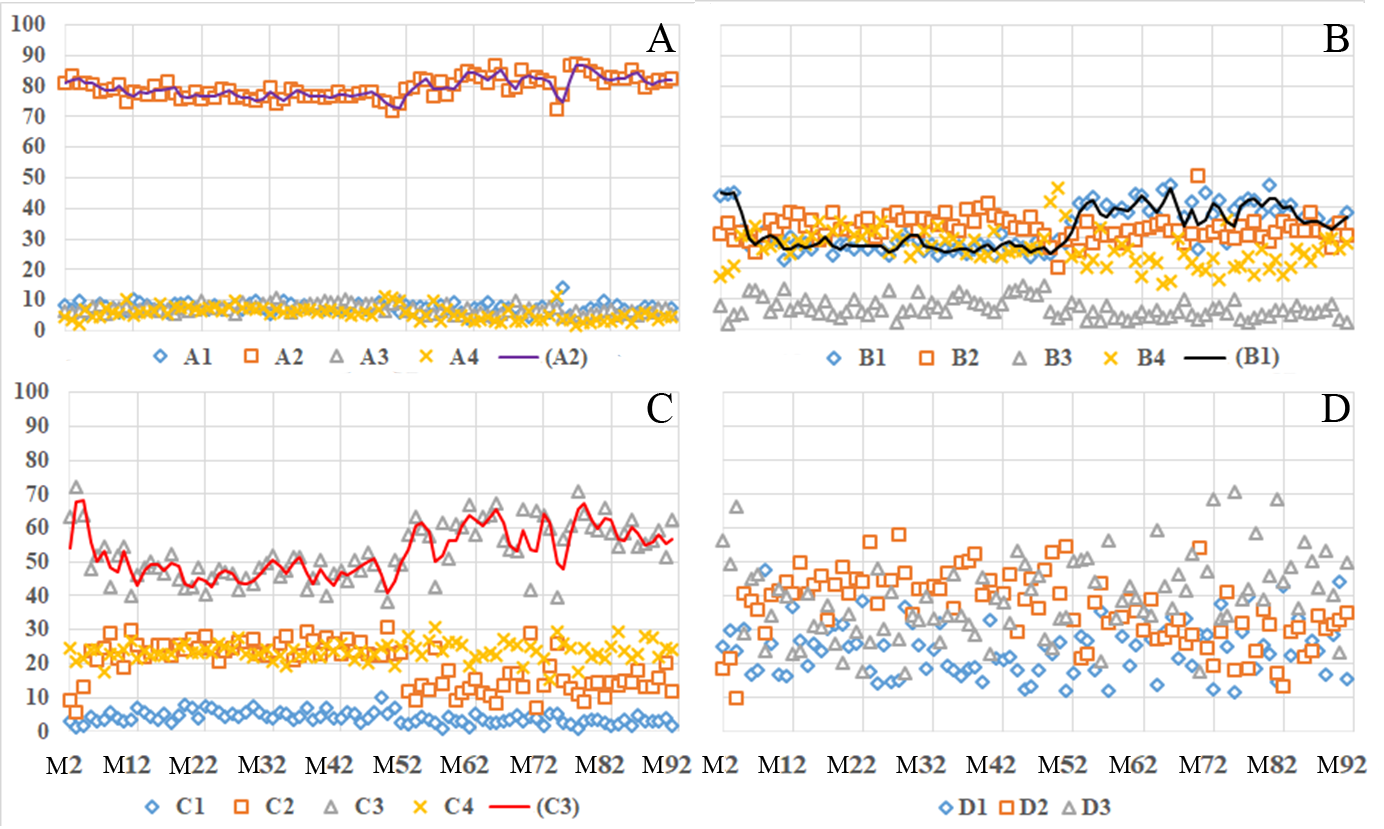

Supplement: S1 Fig — (TIF) [file pone.0235073.s001.tif]

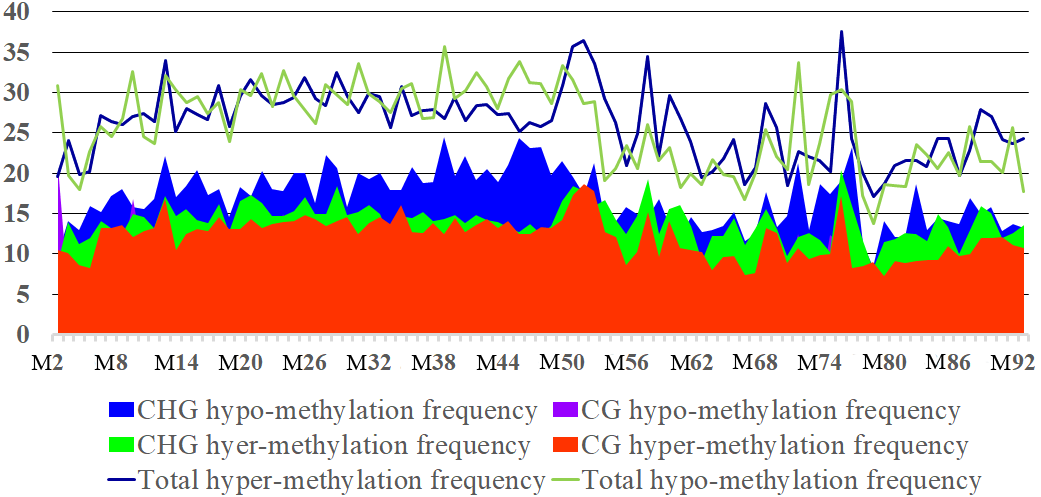

Supplement: S2 Fig — (TIF) [file pone.0235073.s002.tif]
