# Supplementary material for: The assessment of epigenetic diversity, differentiation, and structure in the ‘Fuji’ mutation line implicates roles of epigenetic modification in the occurrence of different mutant groups as well as spontaneous mutants
Source: PLoS One. 2020 Jun 25;15(6):e0235073. doi: 10.1371/journal.pone.0235073 (PMC7316255; doi:10.1371/journal.pone.0235073)
Supplement: S1 Table — (DOCX) [file pone.0235073.s003.docx]

**S1 Table. Adaptors and primer sequences used for preamplification in AFLP and MSAP analyses.**

| **Adaptor and primer** | **Sequence（5'-3'）** | **Adaptor and primer** | **Sequence（5'-3'）** |
| --- | --- | --- | --- |
| *Eco*RI adaptor01 | CTCGTAGACTGCGTACC | *Eco*RI adaptor02 | AATTGGTACGCAGTCTAC |
| *Mse*I adaptor01 | GACGATGAGTCCTGAG | *Mse*I adaptor02 | TACTCAGGACTCAT |
| E00 | GACTGCGTACCAATTC | M00 | GATGAGTCCTGAGTAA |
