# Supplementary material for: The assessment of epigenetic diversity, differentiation, and structure in the ‘Fuji’ mutation line implicates roles of epigenetic modification in the occurrence of different mutant groups as well as spontaneous mutants
Source: PLoS One. 2020 Jun 25;15(6):e0235073. doi: 10.1371/journal.pone.0235073 (PMC7316255; doi:10.1371/journal.pone.0235073)
Supplement: S2 Table — (DOCX) [file pone.0235073.s004.docx]

**S2 Table. Summary of MSAP amplification from 23 primer combinations.**

| **Primer Code** | **Primer Combination** | **Total Loci** | **Number of Methylation-Susceptible Loci  (MSL)** | **Number of polymorphic MSL** | **Percentage of polymorphic MSL (%)** |
| --- | --- | --- | --- | --- | --- |
| 1 | H/M-TCT+E-CT | 136 | 108 | 107 | 99.074 |
| 2 | H/M-TCT+E-CC | 160 | 124 | 114 | 91.935 |
| 3 | H/M-TCT+E-GC | 133 | 77 | 69 | 89.610 |
| 4 | H/M-TCC+E-AG | 156 | 75 | 68 | 90.667 |
| 5 | H/M-TTC+E-AG | 165 | 74 | 64 | 86.486 |
| 6 | H/M-TTC+E-CC | 169 | 114 | 104 | 91.228 |
| 7 | H/M-TTG+E-CA | 121 | 79 | 75 | 94.937 |
| 8 | H/M-TTG+E-CC | 120 | 98 | 88 | 89.796 |
| 9 | H/M-TTA+E-AG | 163 | 69 | 60 | 86.957 |
| 10 | H/M-TTA+E-CA | 144 | 108 | 108 | 100.000 |
| 11 | H/M-TTA+E-CT | 100 | 48 | 48 | 100.000 |
| 12 | H/M-TTA+E-GG | 78 | 42 | 40 | 95.238 |
| 13 | H/M-TGA+E-CT | 158 | 122 | 120 | 98.361 |
| 14 | H/M-TGA+E-CG | 109 | 37 | 30 | 81.081 |
| 15 | H/M-TGA+E-GG | 112 | 75 | 71 | 94.667 |
| 16 | H/M-TGT+E-GG | 59 | 22 | 20 | 90.909 |
| 17 | H/M-TAC+E-CA | 130 | 69 | 65 | 94.203 |
| 18 | H/M-TAC+E-CC | 133 | 57 | 46 | 80.702 |
| 19 | H/M-TAC+E-GC | 106 | 50 | 46 | 92.000 |
| 20 | H/M-TAC+E-GG | 118 | 61 | 56 | 91.803 |
| 21 | H/M-CTC +E-AC | 130 | 77 | 73 | 94.805 |
| 22 | H/M-CTC +E-CG | 137 | 101 | 99 | 98.020 |
| 23 | H/M-CCA +E-CG | 117 | 61 | 56 | 91.803 |
| **Total** |  | 2954 | 1748 | 1627 | 99.074 |
