# Supplementary material for: The assessment of epigenetic diversity, differentiation, and structure in the ‘Fuji’ mutation line implicates roles of epigenetic modification in the occurrence of different mutant groups as well as spontaneous mutants
Source: PLoS One. 2020 Jun 25;15(6):e0235073. doi: 10.1371/journal.pone.0235073 (PMC7316255; doi:10.1371/journal.pone.0235073)
Supplement: S3 Table — (DOCX) [file pone.0235073.s005.docx]

**S3 Table. Summary of AFLP amplification in 92 materials.**

| **Primer combination** | | **The number of Polymorphism loci** | **Total loci** | **Percentage of polymorphism loci (%)** | **Primer combination** | **The number of Polymorphism loci** | **Total loci** | **Percentage of polymorphism loci (%)** |
| --- | --- | --- | --- | --- | --- | --- | --- | --- |
| E-AGG+M-CAA | | 35 | 42 | 83.333 | E-ACC+M-CAG | 111 | 118 | 94.068 |
| E-AGG+M-CAC | | 34 | 36 | 94.444 | E-ACC+M-CTG | 105 | 112 | 93.750 |
| E-AGG+M-CAG | | 49 | 49 | 100.000 | E-ACG+M-CAA | 126 | 141 | 89.362 |
| E-AGG+M-CTG | | 52 | 59 | 88.136 | E-ACG+M-CAC | 41 | 41 | 100.000 |
| E-AGG+M-CTA | | 95 | 95 | 100.000 | E-ACG+M-CAG | 150 | 166 | 90.361 |
| E-AGG+M-CTT | | 101 | 103 | 98.058 | E-ACG+M-CAT | 145 | 148 | 97.973 |
| E-AAG+M-CAC | | 89 | 92 | 96.739 | E-ACG+M-CTG | 51 | 51 | 100.000 |
| E-AAG+M-CAG | | 65 | 89 | 73.034 | E-ACG+M-CTA | 112 | 114 | 98.246 |
| E-ACC+M-CAA | | 28 | 49 | 57.143 | E-ACG+M-CTT | 125 | 130 | 96.154 |
| E-ACC+M-CAC | | 106 | 110 | 96.364 |  |  |  |  |
| **Total** | **1745（Total loci） 1620（Polymorphism loci）** | | | | | | | |
| **Mean** | **86（Polymorphism loci） 92（Total loci）** | | | | | | | |
