# Supplementary material for: The assessment of epigenetic diversity, differentiation, and structure in the ‘Fuji’ mutation line implicates roles of epigenetic modification in the occurrence of different mutant groups as well as spontaneous mutants
Source: PLoS One. 2020 Jun 25;15(6):e0235073. doi: 10.1371/journal.pone.0235073 (PMC7316255; doi:10.1371/journal.pone.0235073)
Supplement: S4 Table — (DOCX) [file pone.0235073.s006.docx]

**S4 Table.** **Variation of the four major types of DNA methylation patterns (CG hypermethylation, CHG hypermethylation, CG hypomethylation, and CHG hypomethylation) in the ‘Fuji’ mutants.**

| **Variety code** | **Hypermethylation** | | | **Hypomethylation** | | |
| --- | --- | --- | --- | --- | --- | --- |
|  | **CHG (%)** | **CG (%)** | **Total** | **CHG (%)** | **CG (%)** | **Total** |
| M2 | 191(9.044) | 222(10.511) | 413(19.555) | 212(10.038) | 439(20.786) | 651(30.824) |
| M3 | 263(13.989) | 189(10.053) | 452(24.043) | 264(14.043) | 106(5.638) | 370(19.681) |
| M4 | 214(11.239) | 164(8.613) | 378(19.853) | 247(12.973) | 96(5.042) | 343(18.015) |
| M5 | 239(11.974) | 165(8.267) | 404(20.240) | 319(15.982) | 133(6.663) | 452(22.645) |
| M6 | 263(14.027) | 247(13.173) | 510(27.200) | 286(15.253) | 197(10.507) | 483(25.760) |
| M7 | 249(13.154) | 250(13.207) | 499(26.360) | 325(17.169) | 140(7.396) | 465(24.564) |
| M8 | 244(12.436) | 266(13.558) | 510(25.994) | 355(18.094) | 170(8.665) | 525(26.758) |
| M9 | 302(14.914) | 246(12.148) | 548(27.062) | 320(15.802) | 341(16.840) | 661(32.642) |
| M10 | 273(14.568) | 240(12.807) | 513(27.375) | 292(15.582) | 168(8.965) | 460(24.546) |
| M11 | 247(13.194) | 248(13.248) | 495(26.442) | 315(16.827) | 129(6.891) | 444(23.718) |
| M12 | 327(17.120) | 321(16.806) | 648(33.927) | 422(22.094) | 191(10.000) | 613(32.094) |
| M13 | 296(14.668) | 211(10.456) | 507(25.124) | 345(17.096) | 267(13.231) | 612(30.327) |
| M14 | 304(15.590) | 242(12.410) | 546(28.000) | 358(18.359) | 202(10.359) | 560(28.718) |
| M15 | 283(14.228) | 260(13.072) | 543(27.300) | 406(20.412) | 181(9.100) | 587(29.512) |
| M16 | 264(13.779) | 246(12.839) | 510(26.618) | 331(17.276) | 195(10.177) | 526(27.453) |
| M17 | 310(16.247) | 278(14.570) | 588(30.818) | 345(18.082) | 203(10.639) | 548(28.721) |
| M18 | 239(12.774) | 244(13.041) | 483(25.815) | 273(14.591) | 175(9.353) | 448(23.944) |
| M19 | 322(16.606) | 253(13.048) | 575(29.654) | 354(18.257) | 235(12.120) | 589(30.376) |
| M20 | 332(17.265) | 275(14.301) | 607(31.565) | 331(17.213) | 239(12.428) | 570(29.641) |
| M21 | 327(16.366) | 264(13.213) | 591(29.580) | 406(20.320) | 240(12.012) | 646(32.332) |
| M22 | 284(14.730) | 265(13.745) | 549(28.475) | 347(17.998) | 199(10.322) | 546(28.320) |
| M23 | 299(14.758) | 283(13.968) | 582(28.727) | 360(17.769) | 304(15.005) | 664(32.774) |
| M24 | 295(15.261) | 273(14.123) | 568(29.384) | 386(19.969) | 187(9.674) | 573(29.643) |
| M25 | 324(17.017) | 283(14.863) | 607(31.880) | 381(20.011) | 151(7.931) | 532(27.941) |
| M26 | 280(14.965) | 267(14.270) | 547(29.236) | 305(16.301) | 185(9.888) | 490(26.189) |
| M27 | 294(14.939) | 265(13.465) | 559(28.404) | 438(22.256) | 171(8.689) | 609(30.945) |
| M28 | 347(18.418) | 265(14.066) | 612(32.484) | 388(20.594) | 172(9.130) | 560(29.724) |
| M29 | 285(14.867) | 280(14.606) | 565(29.473) | 302(15.754) | 244(12.728) | 546(28.482) |
| M30 | 313(15.150) | 257(12.439) | 570(27.590) | 413(19.990) | 282(13.65) | 695(33.640) |
| M31 | 317(16.043) | 273(13.816) | 590(29.858) | 382(19.332) | 208(10.526) | 590(29.858) |
| M32 | 291(15.000) | 282(14.536) | 573(29.536) | 389(20.052) | 171(8.814) | 560(28.866) |
| M33 | 232(11.885) | 268(13.730) | 500(25.615) | 351(17.982) | 187(9.580) | 538(27.561) |
| M34 | 288(14.679) | 315(16.055) | 603(30.734) | 352(17.941) | 246(12.538) | 598(30.479) |
| M35 | 292(14.499) | 256(12.711) | 548(27.210) | 419(20.804) | 208(10.328) | 627(31.132) |
| M36 | 290(15.144) | 241(12.585) | 531(27.728) | 360(18.799) | 153(7.990) | 513(26.789) |
| M37 | 271(14.107) | 266(13.847) | 537(27.954) | 363(18.896) | 155(8.069) | 518(26.965) |
| M38 | 301(14.313) | 263(12.506) | 564(26.819) | 516(24.536) | 235(11.175) | 751(35.711) |
| M39 | 287(14.847) | 280(14.485) | 567(29.333) | 381(19.710) | 184(9.519) | 565(29.229) |
| M40 | 278(13.810) | 257(12.767) | 535(26.577) | 445(22.106) | 165(8.197) | 610(30.303) |
| M41 | 296(14.770) | 273(13.623) | 569(28.393) | 383(19.112) | 268(13.373) | 651(32.485) |
| M42 | 282(14.242) | 283(14.293) | 565(28.535) | 406(20.505) | 203(10.253) | 609(30.758) |
| M43 | 272(13.97) | 258(13.251) | 530(27.221) | 369(18.952) | 177(9.091) | 546(28.043) |
| M44 | 270(13.360) | 284(14.052) | 554(27.412) | 424(20.980) | 218(10.787) | 642(31.766) |
| M45 | 271(12.687) | 267(12.500) | 538(25.187) | 520(24.345) | 202(9.457) | 722(33.801) |
| M46 | 279(13.737) | 254(12.506) | 533(26.243) | 469(23.092) | 165(8.124) | 634(31.216) |
| M47 | 255(12.506) | 271(13.291) | 526(25.797) | 474(23.247) | 161(7.896) | 635(31.143) |
| M48 | 265(13.397) | 261(13.195) | 526(26.593) | 392(19.818) | 174(8.797) | 566(28.615) |
| M49 | 338(16.609) | 290(14.251) | 628(30.860) | 438(21.523) | 242(11.892) | 680(33.415) |
| M50 | 348(18.462) | 326(17.294) | 674(35.756) | 369(19.576) | 228(12.095) | 597(31.671) |
| M51 | 336(17.750) | 354(18.700) | 690(36.450) | 327(17.274) | 215(11.358) | 542(28.632) |
| M52 | 303(15.914) | 338(17.752) | 641(33.666) | 404(21.218) | 146(7.668) | 550(28.887) |
| M53 | 298(16.620) | 227(12.660) | 525(29.281) | 254(14.166) | 89(4.964) | 343(19.130) |
| M54 | 267(14.172) | 229(12.155) | 496(26.327) | 265(14.066) | 122(6.476) | 387(20.541) |
| M55 | 244(12.417) | 169(8.601) | 413(21.018) | 310(15.776) | 150(7.634) | 460(23.410) |
| M56 | 273(14.662) | 191(10.258) | 464(24.919) | 278(14.930) | 105(5.639) | 383(20.569) |
| M57 | 347(19.235) | 274(15.188) | 621(34.424) | 262(14.523) | 208(11.530) | 470(26.053) |
| M58 | 241(12.506) | 185(9.600) | 426(22.107) | 325(16.866) | 91(4.722) | 416(21.588) |
| M59 | 288(15.559) | 261(14.100) | 549(29.660) | 254(13.722) | 176(9.508) | 430(23.231) |
| M60 | 291(16.024) | 196(10.793) | 487(26.817) | 223(12.280) | 107(5.892) | 330(18.172) |
| M61 | 249(13.481) | 194(10.504) | 443(23.985) | 269(14.564) | 100(5.414) | 369(19.978) |
| M62 | 173(9.158) | 194(10.270) | 367(19.428) | 241(12.758) | 111(5.876) | 352(18.634) |
| M63 | 234(12.168) | 155(8.060) | 389(20.229) | 250(13.001) | 167(8.684) | 417(21.685) |
| M64 | 231(12.261) | 180(9.554) | 411(21.815) | 253(13.429) | 120(6.369) | 373(19.798) |
| M65 | 268(14.393) | 182(9.774) | 450(24.168) | 284(15.252) | 81(4.350) | 365(19.603) |
| M66 | 206(11.105) | 138(7.439) | 344(18.544) | 216(11.644) | 94(5.067) | 310(16.712) |
| M67 | 247(13.055) | 145(7.664) | 392(20.719) | 237(12.526) | 143(7.558) | 380(20.085) |
| M68 | 294(15.539) | 249(13.161) | 543(28.700) | 334(17.653) | 147(7.770) | 481(25.423) |
| M69 | 247(13.083) | 237(12.553) | 484(25.636) | 251(13.294) | 166(8.792) | 417(22.087) |
| M70 | 187(9.699) | 170(8.817) | 357(18.517) | 283(14.678) | 111(5.757) | 394(20.436) |
| M71 | 248(12.056) | 220(10.695) | 468(22.752) | 438(21.293) | 256(12.445) | 694(33.738) |
| M72 | 238(12.639) | 177(9.400) | 415(22.039) | 243(12.905) | 108(5.736) | 351(18.640) |
| M73 | 231(11.696) | 195(9.873) | 426(21.57) | 369(18.684) | 99(5.013) | 468(23.696) |
| M74 | 216(10.189) | 212(10.000) | 428(20.189) | 370(17.453) | 262(12.358) | 632(29.811) |
| M75 | 381(20.407) | 321(17.193) | 702(37.600) | 355(19.014) | 213(11.409) | 568(30.423) |
| M76 | 333(15.971) | 173(8.297) | 506(24.269) | 484(23.213) | 119(5.707) | 603(28.921) |
| M77 | 211(11.606) | 155(8.526) | 366(20.132) | 205(11.276) | 108(5.941) | 313(17.217) |
| M78 | 149(8.187) | 163(8.956) | 312(17.143) | 153(8.407) | 97(5.330) | 250(13.736) |
| M79 | 215(11.424) | 137(7.279) | 352(18.704) | 266(14.134) | 84(4.463) | 350(18.597) |
| M80 | 220(11.879) | 169(9.125) | 389(21.004) | 224(12.095) | 118(6.371) | 342(18.467) |
| M81 | 234(12.628) | 165(8.904) | 399(21.533) | 225(12.142) | 114(6.152) | 339(18.295) |
| M82 | 249(12.45) | 182(9.100) | 431(21.550) | 374(18.700) | 97(4.850) | 471(23.550) |
| M83 | 225(11.634) | 178(9.204) | 403(20.838) | 251(12.978) | 180(9.307) | 431(22.285) |
| M84 | 278(15.003) | 172(9.282) | 450(24.285) | 267(14.409) | 114(6.152) | 381(20.561) |
| M85 | 250(13.277) | 207(10.993) | 457(24.270) | 266(14.126) | 158(8.391) | 424(22.517) |
| M86 | 189(10.032) | 183(9.713) | 372(19.745) | 259(13.747) | 113(5.998) | 372(19.745) |
| M87 | 251(12.931) | 193(9.943) | 444(22.875) | 329(16.950) | 171(8.810) | 500(25.760) |
| M88 | 294(15.978) | 220(11.957) | 514(27.935) | 274(14.891) | 121(6.576) | 395(21.467) |
| M89 | 277(15.038) | 220(11.944) | 497(26.982) | 291(15.798) | 104(5.646) | 395(21.444) |
| M90 | 223(12.009) | 225(12.116) | 448(24.125) | 238(12.816) | 135(7.270) | 373(20.086) |
| M91 | 245(12.616) | 216(11.123) | 461(23.738) | 265(13.646) | 234(12.049) | 499(25.695) |
| M92 | 247(13.616) | 194(10.695) | 441(24.311) | 239(13.175) | 83(4.576) | 322(17.751) |
| **Min** | 149(8.187) | 137(7.279) | 312(17.143) | 153(8.407) | 81(4.350) | 250(13.736) |
| **Max** | 381(20.407) | 354(18.700) | 702(37.600) | 520(24.536) | 439(20.786) | 751(35.711) |
| **Mean** | 270(13.993) | 234(12.111) | 504(26.105) | 329(16.954) | 172(8.834) | 501(25.789) |

| **Sample code** | **Hypermethylation** | | | **Hypomethylation** | | |
| --- | --- | --- | --- | --- | --- | --- |
|  | **CHG (%)** | **CG (%)** | **Total** | **CHG (%)** | **CG (%)** | **Total** |
| **2** | 333(15.97) | 173(8.30) | 24.27 | 484(23.21) | 119(5.71) | 28.92 |
| **3** | 211(11.61) | 155(8.53) | 20.13 | 205(11.28) | 108(5.94) | 17.22 |
| **4** | 149(8.19) | 163(8.96) | 17.14 | 153(8.41) | 97(5.33) | 13.74 |
| **5** | 215(11.42) | 137(7.28) | 18.70 | 266(14.13) | 84(4.46) | 18.60 |
| **6** | 220(11.88) | 169(9.13) | 21.00 | 224(12.10) | 118(6.37) | 18.47 |
| **7** | 234(12.63) | 165(8.90) | 21.53 | 225(12.14) | 114(6.15) | 18.29 |
| **8** | 249(12.45) | 182(9.10) | 21.55 | 374(18.70) | 97(4.85) | 23.55 |
| **9** | 225(11.63) | 178(9.20) | 20.84 | 251(12.98) | 180(9.31) | 22.29 |
| **10** | 278(15.00) | 172(9.28) | 24.28 | 267(14.41) | 114(6.15) | 20.56 |
| **11** | 250(13.28) | 207(10.99) | 24.27 | 266(14.13) | 158(8.39) | 22.52 |
| **12** | 189(10.03) | 183(9.71) | 19.75 | 259(13.75) | 113(6.00) | 19.75 |
| **13** | 251(12.93) | 193(9.94) | 22.87 | 329(16.95) | 171(8.81) | 25.76 |
| **14** | 294(15.98) | 220(11.96) | 27.93 | 274(14.89) | 121(6.58) | 21.47 |
| **15** | 277(15.04) | 220(11.94) | 26.98 | 291(15.80) | 104(5.65) | 21.44 |
| **16** | 223(12.01) | 225(12.12) | 24.12 | 238(12.82) | 135(7.27) | 20.09 |
| **17** | 245(12.62) | 216(11.12) | 23.74 | 265(13.65) | 234(12.05) | 25.7 |
| **18** | 247(13.62) | 194(10.69) | 24.31 | 239(13.18) | 83(4.58) | 17.75 |
| **19** | 298(16.62) | 227(12.66) | 29.28 | 254(14.17) | 89(4.96) | 19.13 |
| **20** | 267(14.17) | 229(12.15) | 26.33 | 265(14.07) | 122(6.48) | 20.54 |
| **21** | 244(12.42) | 169(8.60) | 21.02 | 310(15.78) | 150(7.63) | 23.41 |
| **22** | 273(14.66) | 191(10.26) | 24.92 | 278(14.93) | 105(5.64) | 20.57 |
| **23** | 347(19.24) | 274(15.19) | 34.42 | 262(14.52) | 208(11.53) | 26.05 |
| **24** | 241(12.51) | 185(9.60) | 22.11 | 325(16.87) | 91(4.72) | 21.59 |
| **25** | 288(15.56) | 261(14.10) | 29.66 | 254(13.72) | 176(9.51) | 23.23 |
| **26** | 291(16.02) | 196(10.79) | 26.82 | 223(12.28) | 107(5.89) | 18.17 |
| **27** | 249(13.48) | 194(10.50) | 23.98 | 269(14.56) | 100(5.41) | 19.98 |
| **28** | 173(9.16) | 194(10.27) | 19.43 | 241(12.76) | 111(5.88) | 18.63 |
| **29** | 234(12.17) | 155(8.06) | 20.23 | 250(13.00) | 167(8.68) | 21.68 |
| **30** | 231(12.26) | 180(9.55) | 21.82 | 253(13.43) | 120(6.37) | 19.80 |
| **31** | 268(14.39) | 182(9.77) | 24.17 | 284(15.25) | 81(4.35) | 19.60 |
| **32** | 206(11.11) | 138(7.44) | 18.54 | 216(11.64) | 94(5.07) | 16.71 |
| **33** | 247(13.05) | 145(7.66) | 20.72 | 237(12.53) | 143(7.56) | 20.08 |
| **34** | 294(15.54) | 249(13.16) | 28.70 | 334(17.65) | 147(7.77) | 25.42 |
| **35** | 247(13.08) | 237(12.55) | 25.64 | 251(13.29) | 166(8.79) | 22.09 |
| **36** | 187(9.70) | 170(8.82) | 18.52 | 283(14.68) | 111(5.76) | 20.44 |
| **37** | 248(12.06) | 220(10.70) | 22.75 | 438(21.29) | 256(12.45) | 33.74 |
| **38** | 238(12.64) | 177(9.40) | 22.04 | 243(12.90) | 108(5.74) | 18.64 |
| **39** | 231(11.7) | 195(9.87) | 21.57 | 369(18.68) | 99(5.01) | 23.70 |
| **40** | 216(10.19) | 212(10.00) | 20.19 | 370(17.45) | 262(12.36) | 29.81 |
| **41** | 381(20.41) | 321(17.19) | 37.60 | 355(19.01) | 213(11.41) | 30.42 |
| **42** | 191(9.04) | 222(10.51) | 19.55 | 212(10.04) | 439(20.79) | 30.82 |
| **43** | 263(13.99) | 189(10.05) | 24.04 | 264(14.04) | 106(5.64) | 19.68 |
| **44** | 214(11.24) | 164(8.61) | 19.85 | 247(12.97) | 96(5.04) | 18.01 |
| **45** | 239(11.97) | 165(8.27) | 20.24 | 319(15.98) | 133(6.66) | 22.65 |
| **46** | 263(14.03) | 247(13.17) | 27.20 | 286(15.25) | 197(10.51) | 25.76 |
| **47** | 249(13.15) | 250(13.21) | 26.36 | 325(17.17) | 140(7.40) | 24.56 |
| **48** | 244(12.44) | 266(13.56) | 25.99 | 355(18.09) | 170(8.66) | 26.76 |
| **49** | 302(14.91) | 246(12.15) | 27.06 | 320(15.80) | 341(16.84) | 32.64 |
| **50** | 273(14.57) | 240(12.81) | 27.37 | 292(15.58) | 168(8.96) | 24.55 |
| **51** | 247(13.19) | 248(13.25) | 26.44 | 315(16.83) | 129(6.89) | 23.72 |
| **52** | 327(17.12) | 321(16.81) | 33.93 | 422(22.09) | 191(10.00) | 32.09 |
| **53** | 296(14.67) | 211(10.46) | 25.12 | 345(17.10) | 267(13.23) | 30.33 |
| **54** | 304(15.59) | 242(12.41) | 28.00 | 358(18.36) | 202(10.36) | 28.72 |
| **55** | 283(14.23) | 260(13.07) | 27.30 | 406(20.41) | 181(9.10) | 29.51 |
| **56** | 264(13.78) | 246(12.84) | 26.62 | 331(17.28) | 195(10.18) | 27.45 |
| **57** | 310(16.25) | 278(14.57) | 30.82 | 345(18.08) | 203(10.64) | 28.72 |
| **58** | 239(12.77) | 244(13.04) | 25.82 | 273(14.59) | 175(9.35) | 23.94 |
| **59** | 322(16.61) | 253(13.05) | 29.65 | 354(18.26) | 235(12.12) | 30.38 |
| **60** | 332(17.26) | 275(14.30) | 31.57 | 331(17.21) | 239(12.43) | 29.64 |
| **61** | 327(16.37) | 264(13.21) | 29.58 | 406(20.32) | 240(12.01) | 32.33 |
| **62** | 284(14.73) | 265(13.74) | 28.48 | 347(18.00) | 199(10.32) | 28.32 |
| **63** | 299(14.76) | 283(13.97) | 28.73 | 360(17.77) | 304(15.00) | 32.77 |
| **64** | 295(15.26) | 273(14.12) | 29.38 | 386(19.97) | 187(9.67) | 29.64 |
| **65** | 324(17.02) | 283(14.86) | 31.88 | 381(20.01) | 151(7.93) | 27.94 |
| **66** | 280(14.97) | 267(14.27) | 29.24 | 305(16.30) | 185(9.89) | 26.19 |
| **67** | 294(14.94) | 265(13.47) | 28.4 | 438(22.26) | 171(8.69) | 30.95 |
| **68** | 347(18.42) | 265(14.07) | 32.48 | 388(20.59) | 172(9.13) | 29.72 |
| **69** | 285(14.87) | 280(14.61) | 29.47 | 302(15.75) | 244(12.73) | 28.48 |
| **70** | 313(15.15) | 257(12.44) | 27.59 | 413(19.99) | 282(13.65) | 33.64 |
| **71** | 317(16.04) | 273(13.82) | 29.86 | 382(19.33) | 208(10.53) | 29.86 |
| **72** | 291(15.00) | 282(14.54) | 29.54 | 389(20.05) | 171(8.81) | 28.87 |
| **73** | 232(11.89) | 268(13.73) | 25.61 | 351(17.98) | 187(9.58) | 27.56 |
| **74** | 288(14.68) | 315(16.06) | 30.73 | 352(17.94) | 246(12.54) | 30.48 |
| **75** | 292(14.50) | 256(12.71) | 27.21 | 419(20.80) | 208(10.33) | 31.13 |
| **76** | 290(15.14) | 241(12.58) | 27.73 | 360(18.80) | 153(7.99) | 26.79 |
| **77** | 271(14.11) | 266(13.85) | 27.95 | 363(18.90) | 155(8.07) | 26.97 |
| **78** | 301(14.31) | 263(12.51) | 26.82 | 516(24.54) | 235(11.17) | 35.71 |
| **79** | 287(14.85) | 280(14.49) | 29.33 | 381(19.71) | 184(9.52) | 29.23 |
| **80** | 278(13.81) | 257(12.77) | 26.58 | 445(22.11) | 165(8.20) | 30.30 |
| **81** | 296(14.77) | 273(13.62) | 28.39 | 383(19.11) | 268(13.37) | 32.49 |
| **82** | 282(14.24) | 283(14.29) | 28.54 | 406(20.51) | 203(10.25) | 30.76 |
| **83** | 272(13.97) | 258(13.25) | 27.22 | 369(18.95) | 177(9.09) | 28.04 |
| **84** | 270(13.36) | 284(14.05) | 27.41 | 424(20.98) | 218(10.79) | 31.77 |
| **85** | 271(12.69) | 267(12.5) | 25.19 | 520(24.34) | 202(9.46) | 33.80 |
| **86** | 279(13.74) | 254(12.51) | 26.24 | 469(23.09) | 165(8.12) | 31.22 |
| **87** | 255(12.51) | 271(13.29) | 25.80 | 474(23.25) | 161(7.90) | 31.14 |
| **88** | 265(13.40) | 261(13.20) | 26.59 | 392(19.82) | 174(8.80) | 28.61 |
| **89** | 338(16.61) | 290(14.25) | 30.86 | 438(21.52) | 242(11.89) | 33.42 |
| **90** | 348(18.46) | 326(17.29) | 35.76 | 369(19.58) | 228(12.10) | 31.67 |
| **91** | 336(17.75) | 354(18.70) | 36.45 | 327(17.27) | 215(11.36) | 28.63 |
| **92** | 303(15.91) | 338(17.75) | 33.67 | 404(21.22) | 146(7.67) | 28.89 |
| **Total** | **149(9.04)** | **137(8.27)** | **17.14** | **153(10.04)** | **81(5.04)** | **13.74** |
| **Max** | **381(18.46)** | **354(18.70)** | **37.60** | **520(24.54)** | **439(20.79)** | **35.71** |
| **Mean** | **270(14.61)** | **234(13.46)** | **26.11** | **329(18.74)** | **172(10.22)** | **25.79** |
